# YAP/TAZ direct commitment and maturation of lymph node fibroblastic reticular cells

**DOI:** 10.1038/s41467-020-14293-1

**Published:** 2020-01-24

**Authors:** Sung Yong Choi, Hosung Bae, Sun-Hye Jeong, Intae Park, Hyunsoo Cho, Seon Pyo Hong, Da-Hye Lee, Choong-kun Lee, Jin-Sung Park, Sang Heon Suh, Jeongwoon Choi, Myung Jin Yang, Jeon Yeob Jang, Lucas Onder, Jeong Hwan Moon, Han-Sin Jeong, Ralf H. Adams, Jin-Man Kim, Burkhard Ludewig, Joo-Hye Song, Dae-Sik Lim, Gou Young Koh

**Affiliations:** 1 Center for Vascular Research, Institute for Basic Science (IBS), Daejeon, 34141 Republic of Korea; 2 Graduate School of Medical Science and Engineering, Korea Advanced Institute of Science and Technology (KAIST), Daejeon, 34141 Republic of Korea; 3 Biomedical Science and Engineering Interdisciplinary Program, KAIST, Daejeon, 34141 Republic of Korea; 4 Department of Biological Science, KAIST, Daejeon, 34141 Republic of Korea; 5 Department of Otorhinolaryngology, Ajou University School of Medicine, Suwon, 16499 Republic of Korea; 6 Institute of Immunobiology, Kantossipital St. Gallen, St. Gallen, 9007 Switzerland; 7 Department of Otorhinolaryngology - Head and Neck Surgery, Dankook University College of Medicine, Cheonan, 31116 Republic of Korea; 8 Department of Otorhinolaryngology - Head and Neck Surgery, Samsung Medical Center, Sungkyunkwan University School of Medicine, Seoul, 06351 Republic of Korea; 9 Department of Tissue Morphogenesis, Max Planck Institute for Molecular Biomedicine, and Faculty of Medicine, University of Münster, Münster, MD-48149 Germany; 10 Department of Pathology, Chungnam National University School of Medicine, Daejeon, 35015 Republic of Korea

**Keywords:** Developmental biology, Organogenesis, Lymphokines, Lymph node

## Abstract

Fibroblastic reticular cells (FRCs) are immunologically specialized myofibroblasts of lymphoid organ, and FRC maturation is essential for structural and functional properties of lymph nodes (LNs). Here we show that YAP and TAZ (YAP/TAZ), the final effectors of Hippo signaling, regulate FRC commitment and maturation. Selective depletion of YAP/TAZ in FRCs impairs FRC growth and differentiation and compromises the structural organization of LNs, whereas hyperactivation of YAP/TAZ enhances myofibroblastic characteristics of FRCs and aggravates LN fibrosis. Mechanistically, the interaction between YAP/TAZ and p52 promotes chemokine expression that is required for commitment of FRC lineage prior to lymphotoxin-β receptor (LTβR) engagement, whereas LTβR activation suppresses YAP/TAZ activity for FRC maturation. Our findings thus present YAP/TAZ as critical regulators of commitment and maturation of FRCs, and hold promise for better understanding of FRC-mediated pathophysiologic processes.

## Abstract (web summary)

Fibroblastic reticular cells (FRC) are important for lymph node (LN) structure and function. Here the authors show that the YAP/TAZ complex downstream of Hippo signalling regulates FRC commitment and maturation, with YAP/TAZ deficiency impairing FRC differentiation, while hyperactivation of YAZ/TAZ inducing myofibroblastic FRCs and LN fibrosis.

## Introduction

A fine network of fibroblastic reticular cells (FRCs) is essential for maintaining lymph node (LN) structure and function^1–3^. FRCs regulate immune cell entry into the LN via high endothelial venules (HEVs) and confer compartmentalization of lymphocytes within the LN by secretion of essential chemokines^1,4–6^. In this regard, fibrotic damage to FRCs by chronic inflammation and cancer has been shown to seriously deteriorate systemic immune responses by FRCs^7–10^. Thus, proper differentiation of FRC progenitors into mature FRCs during development is critical for acquisition of immunoregulatory characters and initiation of adaptive immune response and chemokine production^1,11–16^.

The differentiation of FRCs involves differentiation of a poorly defined population of mesenchymal cells into FRC precursors, which further develop into mature FRCs^1,12^. Whereas the molecular details involved in the latter process such as lymphotoxin-β receptor (LTβR) and receptor activation of NF-kB ligand (RANKL)-mediated interactions of lymphoid tissue inducer (LTi) cells with FRC precursors have been thoroughly described^17,18^, characterization of stromal cells and signaling pathways involved in the commitment steps of FRC development are incompletely defined^19–21^.

The core of the Hippo pathway consists of large tumor suppressors 1 and 2 (LATS1/2), and yes-associated protein (YAP) and transcriptional co-activator with PDZ-binding motif (TAZ), which are final effectors of the Hippo pathway that exert their functions by mainly interacting with the TEAD/TEF family of transcription factors^22,23^. Upon activation of Hippo pathway, LATS1/2 become phosphorylated and inhibit the activities of YAP and TAZ (YAP/TAZ). This pathway acts as a key regulator of cellular proliferation, differentiation, organ size control, tissue homeostasis, and regeneration^22,24–26^. Despite such diverse and important roles of Hippo pathway in a wide range of biological processes, its role is largely unexplored in LN FRCs.

We hypothesize that the Hippo pathway plays crucial roles in regulating differentiation of FRCs, with relevance to structural and functional properties of LNs. Using FRC-specific genetic targeting and lineage-tracing approaches, we show that YAP/TAZ deficiency impairs FRC differentiation, while hyperactivation of YAZ/TAZ induces myofibroblastic FRCs and LN fibrosis. Thus, we present YAP/TAZ as critical regulators in maintaining FRC integrity and hold promise for better understanding of FRC-mediated physiologic and pathologic conditions.

## Results

### YAP/TAZ support growth and structure of LN by FRCs

To gain insights into the role of the Hippo pathway in LNs, we first examined the expressions and distributions of YAP/TAZ in human and mouse LNs. Both YAP/TAZ were enriched in α-smooth muscle actin (αSMA)^+^ FRCs of healthy human LNs (Supplementary Fig. 1a). Similarly, YAP/TAZ were highly and equally distributed in nucleus and cytoplasm of FRCs, in addition to endothelial cells of high endothelial venules (HEVs) in mouse LNs (Supplementary Fig. 1b).

**Fig. 1:**
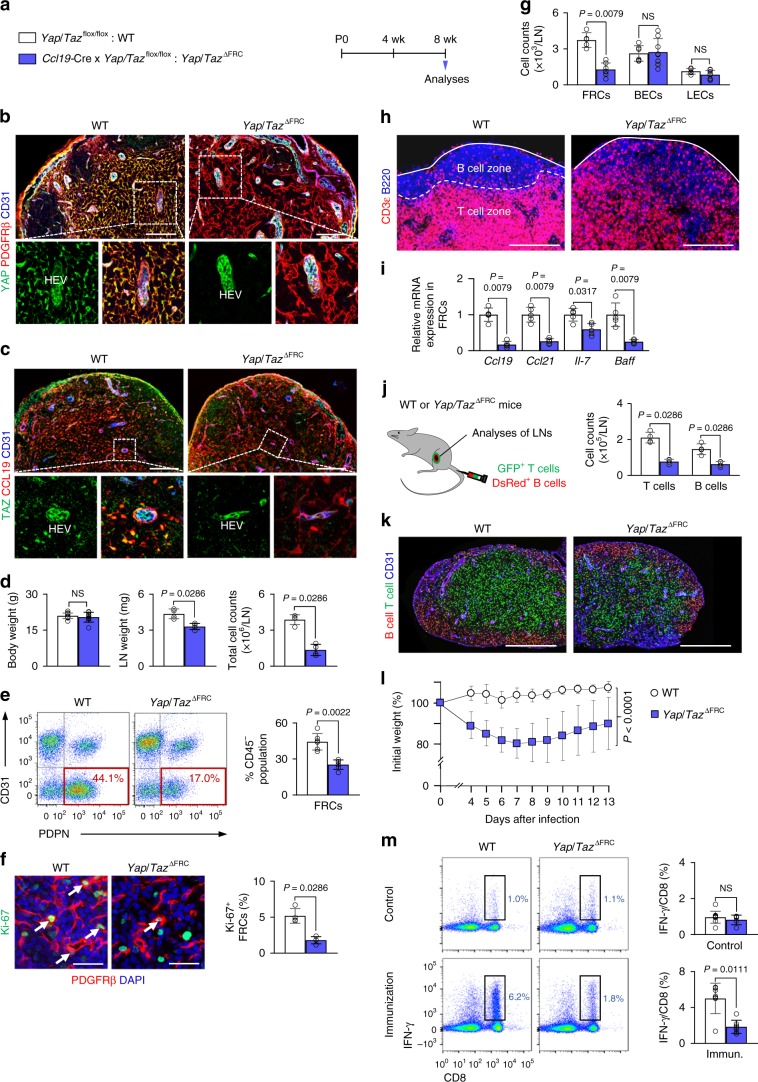
YAP/TAZ support growth and structural organization of LNs by FRCs. **a** Diagram for generation of indicated mice and their analyses at 8 weeks after birth. **b**, **c** Representative images of YAP or TAZ in PDGFRβ^+^ or CCL19^+^ FRCs in WT and *Yap*/*Taz*^∆FRC^ mice. FRCs around high endothelial venule (HEV) within the white dashed-line box are magnified in the lower panels with single-channel YAP or TAZ image. Scale bars, 250 µm. **d** Comparisons of body weight, inguinal LN weight and total number of cells within the inguinal LN in WT (*n* = 11; body weight) and *Yap*/*Taz*^∆FRC^ mice (*n* = 9; body weight). **e** Representative flow cytometric analysis and comparison of proportion of PDPN^+^CD31^−^FRCs (red box) gated from CD45^−^ stromal cells of skin-draining LNs in WT and *Yap*/*Taz*^∆FRC^ mice. **f** Representative images and comparison of Ki-67^+^ FRCs (white arrows) in WT and *Yap*/*Taz*^∆FRC^ mice. Scale bars, 50 µm. **g** Comparison of indicated stromal cell counts gated from CD45^−^ cells of skin-draining LNs in WT and *Yap*/*Taz*^∆FRC^ mice. BECs (*n* = 5), blood endothelial cells; LECs (*n* = 6), lymphatic endothelial cells. **h** Representative images of distinction between B and T cells (white dashed line) beneath the LN capsule (white line) in WT and *Yap*/*Taz*^∆FRC^ mice. Scale bars, 200 µm. **i** Comparison of indicated mRNA expression in FRCs sorted from WT and *Yap*/*Taz*^∆FRC^ mice (quintuplicate values using *n* *=* 10–15 mice/group). **j**, **k** Representative images and comparison of DsRed^+^ B cells and GFP^+^ T cells within the inguinal LN at 24 h after the adoptive transfer in WT and *Yap*/*Taz*^∆FRC^ mice. Scale bars, 500 µm. **l** Changes in body weight after 1 × 10^3^ pfu of A/PR/8 influenza viral infection (*n* = 13). **m** Flow cytometric analyses and comparisons of IFN-γ+CD8^+^ T cells in gated CD3ε^+^ T cells. *n* = 5 (CO) or 7 (IM) mice. Unless otherwise denoted, each dot indicates a value obtained from one mouse and *n* = 4 mice/group pooled from two independent experiments. Horizontal bars indicate mean ± SD and *P* values versus WT by two‐tailed Mann–Whitney *U* test. NS, not significant.

To elucidate the roles of YAP/TAZ in LN FRCs during development, we generated *Yap*/*Taz*^∆FRC^ mice by crossing *Ccl19-*Cre mouse^20^ and *Yap*^flox/flox^^27^/*Taz*^flox/flox^^28^ mouse and analyzed them at 8 weeks after birth (Fig. 1a and Supplementary Fig. 1c). Cre-negative but flox/flox-positive mice among the littermates were defined as wild-type (WT) mice for each experiment. Although we confirmed the depletion of YAP/TAZ in FRCs of *Yap*/*Taz*^∆FRC^ mice, there was no difference in body growth except for a slight reduction in LN weight (Fig. 1b–d). Of note, LNs of *Yap*/*Taz*^∆FRC^ mice revealed reduced total cell number (~64.9%), decreased proportion (~42.9%) and number (~61.1%) of FRCs among CD45^−^ stromal cells, which were less proliferative (~65.2%) but had no difference in apoptosis (Fig. 1d–g and Supplementary Fig. 1d-f). However, LNs of *Yap*/*Taz*^∆FRC^ mice contained similar numbers of BECs and LECs compared with WT (Fig. 1g). To address the selective role of YAP or TAZ in FRCs, we compared LNs of *Yap*^ΔFRC^ or *Taz*^ΔFRC^ mice with those of WT and *Yap*/*Taz*^ΔFRC^ mice (Supplemental Fig. 2a). Of note, decreased LN weight and reduced cellularity observed in *Yap*/*Taz*^ΔFRC^ mice were not evident in *Yap*^ΔFRC^ or *Taz*^ΔFRC^ mice (Supplemental Fig. 2b), suggesting largely redundant roles of YAP and TAZ in FRCs.

Further analysis of LNs revealed that the distinct border between B and T cell zones was disrupted in *Yap*/*Taz*^∆FRC^ mice, but not in *Yap*^ΔFRC^ or *Taz*^ΔFRC^ mice (Fig. 1h and Supplementary Fig. 2c). However, neither proliferation nor apoptosis of immune cells was altered (Supplementary Fig. 3a,b). Instead, analysis of isolated FRCs revealed that mRNA levels of lymphoid chemokines for immune cell trafficking were significantly attenuated in *Yap*/*Taz*^ΔFRC^ mice compared with WT (Fig. 1i and Supplementary Fig. 3c). Indeed, injection of labeled cells showed that the recruitment of transferred GFP^+^ T cells (~63.5%) and DsRed^+^ B cells (~57.0%) were significantly impaired in LNs of *Yap*/*Taz*^∆FRC^ mice compared with WT mice (Fig. 1j, k).

Next, we assessed whether the antiviral immune responses would be affected as a consequence of the changes in *Yap*/*Taz*^∆FRC^ mice by intranasal inoculation of influenza virus (A/PR/8/34). Although there were no apparent differences in immune cell composition in bone marrow, thymus, peripheral circulating blood, and LNs (Supplementary Fig. 4a-i), *Yap*/*Taz*^∆FRC^ mice had less activated interferon-γ (IFN-γ)-secreting effector CD8^+^ T cells, and were severely affected compared with WT mice after challenge with influenza virus (Fig. 1l,m). However, no significant differences were observed in IL-2^+^ CD4^+^ helper T cells and anti-A/PR/8 IgG antibody production between WT and *Yap*/*Taz*^∆FRC^ mice (Supplementary Fig. 5a-c). To better characterize the changes in the FRC phenotype upon *Yap*/*Taz* deletion, we generated WT^FRC-TR^ and *Yap*/*Taz*^∆FRC-TR^ mice by crossing *Yap*/*Taz*^∆FRC^ mouse with *Rosa26-*tdTomato reporter mouse (Supplementary Fig. 6a,b). LNs of *Yap*/*Taz*^∆FRC-TR^ mice revealed increased FRC surface area (~1.5-fold) and ER-TR7^+^ naked conduits (~5.2-fold) compared with WT^FRC-TR^ (Supplementary Fig. 6c,d). In concordance, electron microscope revealed that ~45.8% of conduits were not covered with FRCs and ~30.4% of collagen fibrils were irregularly distributed in the remaining conduits in LNs of *Yap*/*Taz*^∆FRC^ mice compared with WT (Supplementary Fig. 6e,f). However, conduit integrity and functionality were relatively preserved despite the reduced FRC coverage in *Yap*/*Taz*^∆FRC^ mice compared with WT (Supplementary Fig. 6g,h), suggesting that minor structural defects in LN conduit system are unlikely the main cause of altered immune cell trafficking.

### YAP/TAZ hyperactivation impairs differentiation of FRCs

**Fig. 2:**
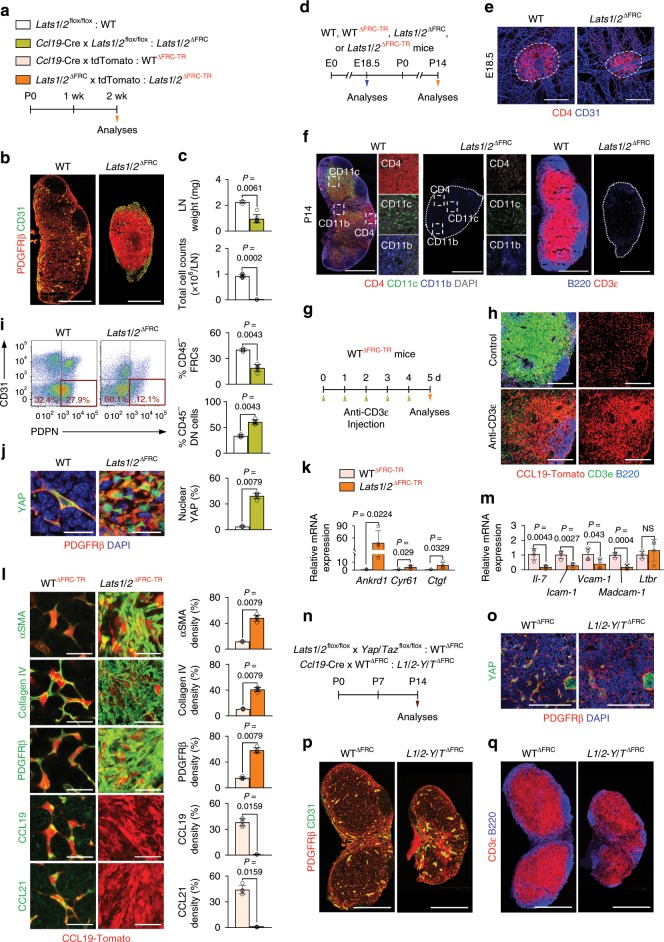
YAP/TAZ hyperactivation impairs differentiation and maturation of FRCs. **a** Diagram for analyses of indicated mice at P14. **b** Representative images of PDGFRβ^+^ FRCs and CD31^+^ vessels in WT and *Lats1*/*2*^∆FRC^ mice (*n* = 5). Scale bars, 500 µm. **c** Comparisons of LN weight (*n* = 4–7) and total number of cells (*n* = 6–10) in WT and *Lats1*/*2*^∆FRC^ mice. **d** Diagram for analyses of indicated mice at E18.5 or P14. **e** Representative images of LN anlagen (dashed line) at E18.5 showing CD4^+^ LTi cells in WT and *Lats1*/*2*^ΔFRC^ mice (*n* = 6). Scale bars, 200 μm. **f**, Representative images of indicated markers (dashed box) within the inguinal LN (dotted-line) in WT and *Lats1*/*2*^ΔFRC^ mice at P14 (*n* = 6). Scale bars, 500 µm. **g**, **h** Diagram and representative images for analyses of WT^ΔFRC-TR^ mice (*n* = 6) that were injected with anti-CD3ε for 5 days to induce T cell depletion. Scale bars, 100 μm. **i** Representative flow cytometric plots and comparison of proportion of PDPN^+^CD31^−^ FRCs (red box) and PDPN^−^CD31^−^ double-negative (DN) cells of skin-draining LNs in WT and *Lats1*/*2*^∆FRC^ (*n* = 5–6) mice. **j** Representative images and comparison of YAP expression and nuclear localization (green-arrowheads) in LN of WT and *Lats1*/*2*^∆FRC^ mice (*n* = 5). Scale bars, 20 µm. **k** Comparison of indicated mRNA expression in FRCs sorted from WT^ΔFRC-TR^ and *Lats1*/*2*^iΔFRC-TR^ mice (*n* = 4). **l** Representative images and comparisons of indicated marker expressions in LNs of WT and *Lats1*/*2*^∆FRC^ mice (*n* = 4–5). Scale bars, 20 µm. **m** Comparison of indicated mRNA expression in FRCs sorted from WT^ΔFRC-TR^ and *Lats1*/*2*^iΔFRC-TR^ mice. **n** Diagram for analyses of indicated mice at P14. **o** Representative images of YAP expression in LNs of WT and *L1*/*2-Y*/*T*^∆FRC^mice. Scale bars, 100 µm. **p**, **q** Representative images of indicated markers in LNs of WT and *L1*/*2-Y*/*T*^∆FRC^ mice. Scale bars, 500 µm. Unless otherwise denoted, each dot indicates a value obtained from inguinal LN and *n* = 4 mice. Horizontal bars indicate mean ± SD and *P* values versus WT or WT^ΔFRC-TR^ by two‐tailed Mann‐Whitney *U* test except for (**k**) and (**m**) (two-tailed Student’s *t*-test). NS, not significant.

To specifically hyperactivate YAP/TAZ in FRCs during LN development, we generated *Lats1*/*2*^∆FRC^ mutants by crossing *Ccl19-*Cre mouse and *Lats1*^flox/flox^ ^29^/*Lats2*^flox/flox^ ^30^ mouse (Fig. 2a). Neonatal *Lats1*/*2*^∆FRC^ mice exhibited substantial growth retardation with lethality at 16–21 days after birth (Supplementary Fig. 7a-c). Moreover, they showed severely disrupted structural organization of LNs with impaired lymphatic drainage (Fig. 2b and Supplementary Fig. 7d,e). LN weight (~61%), total number of cells within the LN (~98%), and the fraction of stromal cell populations of LNs were also markedly reduced in *Lats1*/*2*^∆FRC^ mice compared with WT (Fig. 2c and Supplementary Fig. 7f).

Since immune cells reorganize the reticular network after birth^3^, we examined the recruitment of lymphoid tissue inducer (LTi) cells and the organization of lymphoid tissue organizer (LTo) cells both at the embryonic and postnatal period in *Lats1*/*2*^∆FRC^ mice (Fig. 2d).

At embryonic (E) day 14.5, engagement of LECs and CD4^+^ LTi cells were observed in WT and *Lats1*/*2*^ΔFRC^ embryos (Supplementary Fig. 7g), which led to preserved recruitment of LTi cells at E18.5 (Fig. 2e). Nevertheless, substantial defects in organization of LTo cells were observed at E18.5 and postnatal day (P)5 in both *Yap*/*Taz*^∆FRC^ and *Lats1*/*2*^∆FRC^ mice compared with WT mice (Supplemental Fig. 7h-j). Thus, adequate activation of YAP/TAZ in FRCs is required for LN development. Moreover, the distinct border between B and T cell zones was disrupted in both *Yap*/*Taz*^∆FRC^ and *Lats1*/*2*^∆FRC^ mice compared with WT mice at P7 (Supplemental Fig. 7k), whereas immune cells were rarely observed in growing LNs of *Lats1*/*2*^∆FRC^ neonates compared with WT neonates at P14, implying that immune cell recruitment was impaired during the postnatal period in *Lats1*/*2*^ΔFRC^ mice (Fig. 2f). To evaluate whether the apparent dense distribution of FRCs was due to the reduced number of immune cells, we depleted T lymphocytes^31^ and other immune cells^32^ by daily injection of anti-CD3ε mAb into WT^∆FRC-TR^ mice (Fig. 2g). Of note, while T cells were depleted, increase in FRC density was evident in anti-CD3ε mAb-injected mice compared with control mice (Fig. 2h), indicating that impaired immune cell recruitment could conversely affect the density of FRCs during development.

Further analysis of LNs in *Lats1*/*2*^∆FRC^ mice at P14 showed a reduction in FRCs (~52%) but an increase in PDPN^−^/CD31^−^ double-negative (DN) populations (~1.8-fold) compared with WT mice (Fig. 2i). To investigate whether LN phenotypes of *Lats1*/*2*^∆FRC^ mice are caused by impaired differentiation of FRCs, we performed lineage-tracing assay using *Lats1*/*2*^∆FRC-TR^ mice, generated by crossing *Lats1*/*2*^∆FRC^ mouse with *Rosa26-*tdTomato reporter mouse (Fig. 2a). We confirmed enhanced nuclear localization of YAP with upregulated YAP target genes in LN FRCs of *Lats1*/*2*^∆FRC^ mice and Lats1/*2*^∆FRC-TR^ mice compared with their controls (Fig. 2j, k). Indeed, we found high expression of Tomato in PDGFRβ^+^ cells within the LNs of *Lats1*/*2*^∆FRC-TR^ mice, indicating that these cells arose from FRC precursors (Supplementary Fig. 7l). In addition, Tomato^+^ FRCs of *Lats1*/*2*^∆FRC-TR^ mice highly expressed αSMA, collagen IV, and PDGFRβ, which are canonical myofibroblastic markers of FRCs but also direct targets of YAP/TAZ^33^ (Fig. 2l). In contrast, expressions of markers of differentiated FRCs including CCL19 and CCL21 were markedly attenuated in FRCs of *Lats1*/*2*^∆FRC-TR^ mice compared with WT mice (Fig. 2l, m), suggesting that YAP/TAZ hyperactivation impairs FRC differentiation during development.

Because LATS1/2 can target several pathways^25^, we sought to ascertain if YAP/TAZ are indeed the pivotal target responsible for the aforementioned phenotypes. In this regard, we generated *L1*/*2-Y*/*T*^∆FRC^ mice by crossing *Lats1*/*2*^∆FRC^ and *Yap*/*Taz*^∆FRC^ mice (Fig. 2n, o). Of note, the vessels within the LN and the distinct B and T cell zones were partially restored in *L1*/*2-Y*/*T*^∆FRC^ mice compared with WT mice (Fig. 2p, q), implying that YAP/TAZ are major targets of LATS1/2 in FRC differentiation during LN development.

### YAP/TAZ are dispensable in adult mature FRCs

**Fig. 3:**
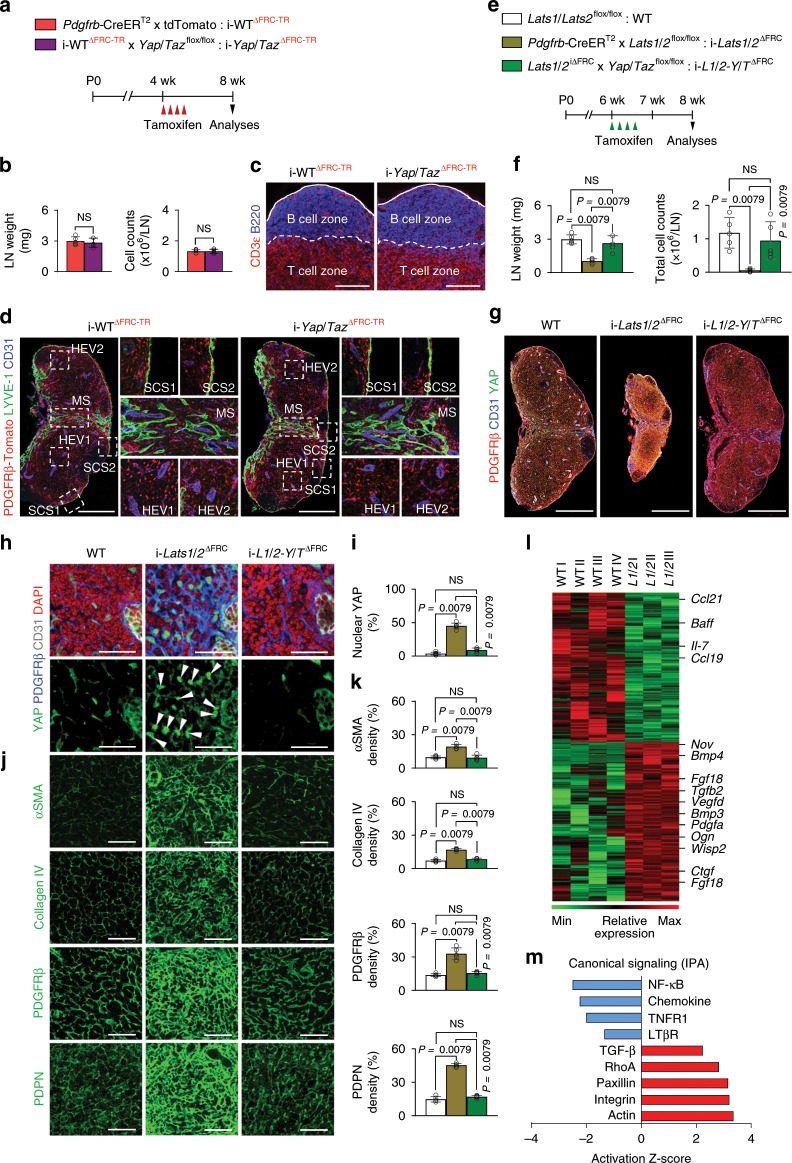
Canonical Hippo pathway LATS1/2-YAP/TAZ governs FRCs. **a** Diagram for generation of indicated mice and their analyses at 8-weeks old after the tamoxifen injection from 4-weeks old. **b** Comparisons of the inguinal LN weight and cellularity within the inguinal LN in i-WT^ΔFRC-TR^ and i-*Yap*/*Taz*^ΔFRC-TR^ mice. **c** Representative images of intact border between B and T cell zones (white dashed line) beneath the LN capsule (white line) in i-WT^ΔFRC-TR^ and i-*Yap*/*Taz*^ΔFRC-TR^ mice (*n* = 4). Scale bars, 200 μm. **d** Representative images of preserved LYVE-1^+^ lymphatic vessels and CD31^+^ blood vessels within the inguinal LN in i-WT^ΔFRC-TR^ and i-*Yap*/*Taz*^ΔFRC-TR^ mice (*n* = 4). The regions within the white dashed-line box around subcapsular sinuses (SCS), medullary sinus (MS) and HEVs are magnified as indicated. Scale bars, 500 μm. **e** Diagram for generation of indicated mice for their analyses at 8-weeks old after the tamoxifen delivery from 6-weeks old. **f** Comparisons of the inguinal LN weight and total number of cells within the inguinal LN in WT, i-*Lats1*/*2*^∆FRC^ or i-*L1*/*2-Y*/*T*^∆FRC^ mice. **g** Representative images of inguinal LN in WT, i-*Lats1*/*2*^∆FRC^ or i-*L1*/*2-Y*/*T*^∆FRC^ mice. Scale bars, 500 μm. **h**, **i** Representative images and comparison of YAP nuclear localization (white arrowheads) in inguinal LN of WT, i-*Lats1*/*2*^∆FRC^ or i-*L1*/*2-Y*/*T*^∆FRC^ mice. Scale bars, 40 µm. **j**, **k** Representative images and comparisons of indicated marker expressions in FRCs around T cell zone of inguinal LN in WT, i-*Lats1*/*2*^∆FRC^ or i-*L1*/*2-Y*/*T*^∆FRC^ mice. Scale bars, 60 µm. **l** Heatmap and hierarchical clustering of differentially expressed genes of RNA-Seq data in isolated FRCs from WT and i-*Lats1*/*2*^∆FRC^ mice and list of selected downregulated genes (green) encoding cytokines and chemokines and upregulated genes (red) involved in TGF-β signaling. **m** Canonical IPA-annotated pathways listed in absolute IPA activation Z-score (*P* < 0.05) to identify potential activation or inhibition of indicated signaling pathways in isolated FRCs from i-*Lats1*/*2*^∆FRC^ mice compared with WT. Unless otherwise denoted, each dot indicates a value obtained from one mouse and *n* = 5 mice/group pooled from two independent experiments. Horizontal bars indicate mean ± SD and *P* values versus WT, i-WT^ΔFRC-TR^ or i-*Lats1*/*2*^∆FRC^ by two‐tailed Mann‐Whitney *U* test. NS, not significant.

To uncover the roles of YAP/TAZ in adult mature LN FRCs, we generated i-*Yap*/*Taz*^∆FRC^ mice by crossing *Pdgfrb*-CreER^T2^ mouse, for which we confirmed high Cre activity (~85%) in PDGFRβ^+^ FRCs (Supplementary Fig. 8a-c), with *Yap*^flox/flox^/*Taz*^flox/flox^ mouse and administered tamoxifen to 4-weeks old mice and analyzed them after 4 weeks (Fig. 3a). No apparent differences were observed in weight, cellularity, chemokine expression, border of B and T cell zones and distribution of lymphatic vessels in inguinal LNs of *Yap*/*Taz*^i∆FRC^ mice compared with WT mice (Fig. 3b–d and Supplementary Fig. 8d), implying that YAP/TAZ are dispensable for adult mature LN FRCs.

To examine whether the canonical LATS1/2-YAP/TAZ pathway also operates during adulthood, we generated i-*L1*/*2-Y*/*T*^∆FRC^ mice by crossing *Lats1*^flox/flox^/*Lats2*^flox/flox^ mouse with i-*Yap*/*Taz*^∆FRC^ mouse, and administered tamoxifen to 6-weeks-old mice and analyzed them 2 weeks later (Fig. 3e). Of note, size, weight, cellularity and distribution of PDGFRβ^+^ FRCs in inguinal LNs of *L1*/*2-Y*/*T*^i∆FRC^ mice were comparable to those of WT mice (Fig. 3f, g). Furthermore, although expressions of αSMA, collagen IV, PDGFRβ, and PDPN in LN FRCs were increased in i-*Lats1*/*2*^∆FRC^ mice, those in i-*L1*/*2-Y*/*T*^∆FRC^ mice were comparable to WT mice (Fig. 3h–k). In support of this notion, our ATAC-sequencing analysis showed enrichment of *Pdpn* promoter region upon *Lats1*/*2* deletion, which, on the other hand, was abrogated upon *Yap*/*Taz* deletion (Supplementary Fig. 9a). These results indicate that YAP/TAZ activation must be controlled to maintain the homeostasis of LNs during adulthood.

### Identification of YAP/TAZ-regulated pathways in FRCs

To gain insights into roles of YAP/TAZ in FRC differentiation and maturation, we performed transcriptomic analysis of gene expression profiles in isolated FRCs from LNs of WT and *Yap*/*Taz*^∆FRC^ mice. Gene Set Enrichment Analysis (GSEA) disclosed significant reductions in genes regulating the epithelial-mesenchymal transition (EMT) and E2F target genes together with reductions in YAP target genes in FRCs of *Yap*/*Taz*^∆FRC^ compared with WT (Supplementary Fig. 9b,c). Further analysis of FRCs from LNs of i-*Lats1*/*2*^∆FRC^ mice showed that genes related to EMT and TGF-β signaling were enriched together with upregulation of YAP targets compared with WT mice (Supplementary Fig. 9d,e). Consistently, TGF-β signaling-associated genes such as *Bmp4*, *Bmp3*, *Tgfb2*, and *Ogn* and fibrosis-associated genes including *Wisp2*, *Nov*, *Fgf18*, *Ctgf*, and *Pdgfa* were ranked among the top 10 upregulators by YAP/TAZ hyperactivation in FRCs (Fig. 3l and Supplementary Fig. 9f). Indeed, genes related with extracellular matrix (ECM), Rho signaling and actin-cytoskeleton rearrangement were upregulated, while those encoding cytokines and chemokines including *Ccl19*, *Ccl21*, *Il-7*, and *Baff* were downregulated in FRCs of i-*Lats1*/*2*^∆FRC^ mice compared with WT mice (Fig. 3l, m and Supplementary Fig. 9g). Ingenuity Pathway Analysis (IPA) revealed that the signaling pathways related to both canonical and non-canonical NF-κB signaling and production of chemokines were downregulated in LN FRCs of *Lats1*/*2*^i∆FRC^ mice compared with WT mice (Fig. 3m).

### YAP/TAZ hyperactivation in FRCs impedes immune response

To assess whether defects in FRC differentiation influence adaptive immune response in i-*Lats1*/*2*^∆FRC^ mice, we first examined the expressions of homeostatic chemokines and lymphocyte survival factors in FRCs^6,20^. Lower mRNA levels of *Ccl19*, *Ccl21*, *Il-7*, and *Baff* in addition to lower levels of CCL19 and CCL21 were found in LN FRCs of i-*Lats1*/*2*^∆FRC^ mice compared with WT mice (Supplementary Fig. 10a-d). Although the structures of HEVs were preserved, homing of labeled-lymphocytes into the LNs of i-*Lats1*/*2*^∆FRC^ mice was markedly impaired after the adoptive transfer (Supplementary Fig. 10e-i). When we performed adoptive transfer with CFSE-labeled OT-II CD4^+^ T cells followed by immunization with ovalbumin (OVA) via footpad injection, both proliferation and activation of OT-II CD4^+^ T cells were impaired at 3 days after the OVA injection in LNs of i-*Lats1*/*2*^∆FRC^ mice compared with those of WT mice (Supplementary Fig. 10j-l). Nevertheless, neither alteration in production or differentiation of lymphocytes in bone marrow nor proportion of immune cells including T and B lymphocytes in LNs were found (Supplementary Fig. 11a-d). Thus, defects in FRC differentiation by YAP/TAZ hyperactivation primarily results in impaired adaptive immune response.

### YAP/TAZ are activated in myofibroblastic FRC precursors

**Fig. 4:**
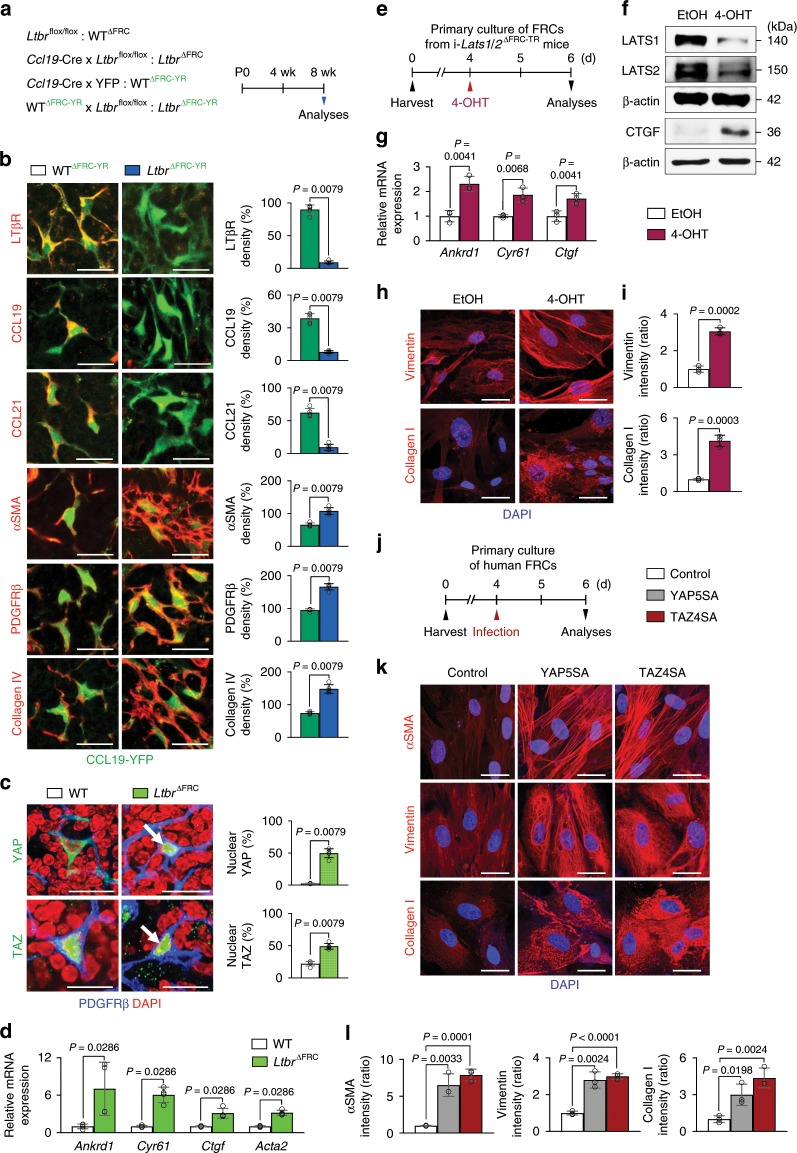
FRC-specific depletion of Ltbr activates YAP/TAZ-induced myofibrosis. **a** Diagram for generation of indicated mice and their analyses at 8-weeks old. **b** Representative images and comparisons of indicated marker expressions on CCL19-YFP^+^ FRCs in WT^∆FRC-YR^ and *Ltbr*^∆FRC-YR^ mice. Scale bars, 20 µm. **c** Representative images and comparisons of YAP and TAZ nuclear localization (white arrows) in inguinal LN of WT^∆FRC^ and *Ltbr*^∆FRC^ mice. Scale bars, 20 µm. **d** Comparison of indicated mRNA expression in FRCs sorted from WT^∆FRC^ and *Ltbr*^∆FRC^ mice. Each dot indicates a mean of quadruplicate values using *n* *=* 8–12 mice/group from three independent experiments. **e** Diagram for primary culture of FRCs derived from i-*Lats1*/*2*^∆FRC-TR^ mice and treatment with EtOH (control) or 4-OHT at 4 days after the culture and their analyses at 2 days after the treatment. **f** Immunoblot analysis of indicated proteins in primary cultured mouse FRCs after treatment with EtOH or 4-OHT for 2 days. **g** Comparisons of indicated mRNA expression normalized to *Gapdh* in primary cultured mouse FRCs after treatment with EtOH or 4-OHT for 2 days. Each dot indicates a mean of triplicate values from three independent experiments. **h**, **i** Representative images and comparisons of indicated marker expressions in primary cultured mouse FRCs after treatment with EtOH or 4-OHT for 2 days. Scale bars, 30 μm. Each dot indicates a mean of triplicate values from three independent experiments. **j** Diagram for primary culture of human FRCs for 4 days and infection with an adenovirus to induce overexpression of active YAP (YAP5SA) or TAZ (TAZ4SA) for their analyses at 2 days after the infection. **k**, **l** Representative images and comparisons of indicated marker expressions in primary cultured human FRCs infected with control-, YAP5SA-, or TAZ4SA-adenovirus. Scale bars, 30 μm. Each dot indicates a mean of triplicate values from three independent experiments. Unless otherwise denoted, each dot indicates a value obtained from one mouse and *n* = 5 mice/group pooled from two independent experiments. Horizontal bars indicate mean ± SD and *P* values versus WT^∆FRC^ or WT^∆FRC-YR^ by two‐tailed Mann‐Whitney *U* test except for (**g**), (**i**), and (**l**) (two-tailed Student’s *t*-test). NS, not significant.

Of note, LTβR signaling, which is known to regulate FRC maturation^20,21^, was predicted to be preferentially and significantly influenced by YAP/TAZ hyperactivation in FRCs by our IPA analysis (Fig. 3m). We therefore sought to corroborate the interaction between LTβR and YAP/TAZ in FRCs by generating *LTbR*^∆FRC-YR^ mice by crossing *Ccl19*-Cre mouse with *Ltbr*^flox/flox^ and YFP reporter mouse (Fig. 4a). Consistent with previous reports^20,21^, levels of αSMA, PDGFRβ and collagen IV in FRCs were increased, while levels of LTβR, CCL19 and CCL21 were decreased in *Ltbr*^∆FRC^ mice compared with those of WT mice (Fig. 4b). Importantly, YAP/TAZ were nuclear localized and expressions of YAP target genes were upregulated in FRCs of *Ltbr*^∆FRC^ mice compared with WT mice (Fig. 4c, d), suggesting that YAP/TAZ activity could be negatively regulated by LTβR signaling.

To evaluate whether YAP/TAZ hyperactivation-induced myofibroblastic phenotypes in vivo are mediated through FRC-intrinsic signaling, we cultured primary FRCs derived from i-*Lats1*/*2*^∆FRC-TR^ mice. Following 4-hydroxytamoxifen (4-OHT) treatment, efficient (~83.7%) depletion of LATS1/2 and upregulation of YAP target genes were confirmed compared with control EtOH treatment (Fig. 4e–g and Supplementary Fig. 12a,b). In this condition, levels of pro-fibrotic markers such as vimentin and collagen I^34^ were increased, and contractility was potentiated as shown as enhanced assembly of F-actin (Fig. 4h, i and Supplementary Fig. 12c). To see whether this finding can be recapitulated in human, we cultured primary FRCs derived from human normal LNs. Primary cultured human FRCs transfected with adenovirus encoding constitutive active form of YAP (YAP5SA) or TAZ (TAZ4SA) enhanced the levels of αSMA, vimentin and collagen I compared with those transfected with control vector (Fig. 4j–l), indicating that phenotypes of YAP/TAZ hyperactivation in FRCs are instrinsic.

### YAP/TAZ regulate chemokine expression before LTβR engagement

**Fig. 5:**
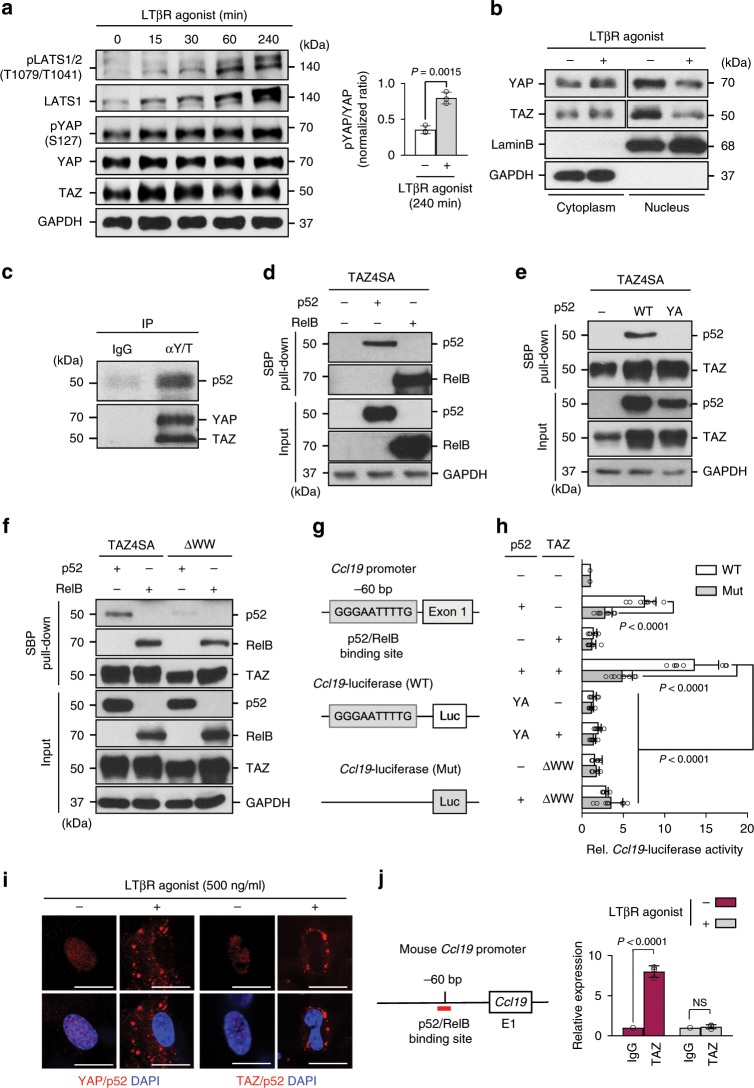
YAP/TAZ regulate chemokine expression prior to LTβR engagement. **a** Immunoblot analyses at indicated time points and comparison of normalized pYAP/YAP ratio at 240 min in cultured FRCs derived from WT mice after stimulation with LTβR agonistic antibody (500 ng/ml) for indicated time points. **b** Immunoblot analyses of indicated proteins in nuclear (LaminB) and cytoplasmic (GAPDH) fractions of cultured FRCs after treatment with or without LTβR agonistic antibody. **c** Immunoprecipitation (IP) with anti-IgG or anti-YAP/TAZ (αY/T) antibody in primary cultured FRCs derived immunoblot with indicated antibodies. **d** Pull-down assay with streptavidin resin in HEK-293T cells after transfection with the streptavidin-binding peptide (SBP)-TAZ4SA, with or without plasmids encoding p52 or RelB and immunoblot analysis with indicated antibodies. **e** Pull-down assay with streptavidin resin in HEK-293T cells after transfection with the (SBP)-TAZ4SA, with or without plasmids encoding p52 (WT) or p52-Y293A mutants (YA) and immunoblot analysis with indicated antibodies. **f** Pull-down assay with streptavidin resin in HEK-293T cells after transfection with (SBP)-TAZ4SA or (SBP)-WW domain-deleted TAZ mutant (△WW) with or without plasmids encoding p52 or RelB and immunoblot analysis with indicated antibodies. **g** Diagram depicting the p52/RelB binding site within the mouse *Ccl19* promoter and *Ccl19* promoter-driven luciferase constructs containing p52/RelB binding site (WT) or the binding site deletion mutant (Mut). **h** Comparison of relative luciferase reporter activity using WT and Mut in HEK-293T cells. WT and Mut was co-transfected with or without p52 or p52 mutant (YA) and TAZ or TAZ mutant (△WW) in HEK-293T cells (*n* = 8). *P* values by one-way ANOVA. **i** Representative images of in situ proximity ligation assay showing localizations of YAP or TAZ and p52 after treatment with or without LTβR agonistic antibody in cultured FRCs. Nuclei are stained with DAPI. Scale bars, 50 µm. **j** ChIP experiments using IgG or anti-TAZ antibody were performed in MEFs infected with retrovirus encoding CTL or TAZ4SA with or without LTβR agonistic antibody. Unless otherwise denoted, similar findings were observed in three independent experiments. Horizontal bars indicate mean ± SD and *P* value versus 0 min or Control by two-tailed Student’s *t*-test. NS, not significant.

To examine the regulatory mechanism between LTβR and YAP/TAZ, primary cultured FRCs derived from WT mice were stimulated with an agonistic LTβR antibody (Supplementary Fig. 13a,b). As expected, the stimulation of LTβR increased NIK protein but reduced p100 protein in time and dose-dependent manners (Supplementary Fig. 13b,c). Of note, it not only increased p52 protein level, but also promoted activity of LATS and increased pYAP/YAP ratio (Fig. 5a). Importantly, nuclear‐cytoplasmic fractionation and immunofluorescence analyses revealed that the LTβR stimulation promoted nuclear to cytoplasmic shuttling of YAP/TAZ (Fig. 5b and Supplementary Fig. 13d). Thus, LTβR signaling suppresses YAP/TAZ activity in FRCs.

LTβR intracellular signaling mainly takes a non-canonical NF-κB pathway through p52/RelB to exert its cellular functions^35,36^. To examine whether YAP/TAZ physically interact with either p52 or RelB, we performed immunoprecipitation analysis using anti-YAP/TAZ antibody in primary cultured FRCs derived from WT mice. p52 was readily detected in the immuno-complexes pulled with anti-YAP/TAZ antibody but not in those from control IgG (Fig. 5c). To ensure this finding, we transfected HEK293T cells with a gene encoding streptavidin-binding peptide-tagged constitutively active form of TAZ (SBP-TAZ4SA) and pulled all bound proteins using the streptavidin resin. We confirmed presence of both p52 and RelB in the resin, and stabilization of p52 protein through TAZ (Fig. 5d and Supplementary Fig. 13e).

YAP/TAZ contain WW domains that bind to PPxY motif of the binding protein partners^25,37^. Although neither p52 nor RelB has PPxY motif, both p52 and RelB have highly conserved PPY motifs^38,39^, which could be possible binding targets for YAP/TAZ. Of note, point mutation of the PPY motif of p52 to PPA (p52-Y293A) completely abrogated the binding interaction with SBP-TAZ4SA (Fig. 5e), whereas the RelB mutants (RelB-Y248A and RelB-Y341A) still showed interaction with SBP-TAZ4SA (Supplementary Fig. 13f). Conversely, deleting the WW domain in SBP-TAZ4SA (TAZ4SA△WW) markedly attenuated its binding with p52, but again this did not affect the binding with RelB (Fig. 5f), indicating that TAZ independently binds to both RelB and p52. To elucidate how this molecular interaction regulates FRC differentiation, we transfected HEK293T cells retaining a *Ccl19* promoter-driven luciferase with the gene encoding either TAZ4SA, TAZ4SA△WW, p52, or p52-Y293A (Fig. 5g). Intriguingly, while a single transfection of p52 enhanced the luciferase activity by 7.5-fold, co-transfection with TAZ4SA and p52 promoted the luciferase activity by 13.5-fold (Fig. 5h). However, this increase in luciferase activity was not observed in cells that were transfected with either TAZ4SA△WW, p52-Y293A or both of these (Fig. 5h). We further validated our findings in primary cultured FRCs derived from i-*Yap*/*Taz*^∆FRC^ mice, where p52-regulated transcripts such as *Ccl19* and *Ccl21* were markedly attenuated by 4-OHT treatment compared with EtOH (Supplementary Fig. 13g,h). Conversely, in situ proximity ligation assay and chromatin immunoprecipitation (ChIP) analysis revealed that LTβR stimulation promoted nuclear to cytoplasmic shuttling of YAP/TAZ-p52 complex and attenuated its binding affinity to promoter regions of *Ccl19* (Fig. 5i, j). Thus, proper interaction between YAP/TAZ and p52 seems to be required for the expression of chemokines such as *Ccl19* before LTβR engagement (Supplementary Fig. 13i).

### YAP/TAZ drive specification of mesenchymal cells into FRCs

**Fig. 6:**
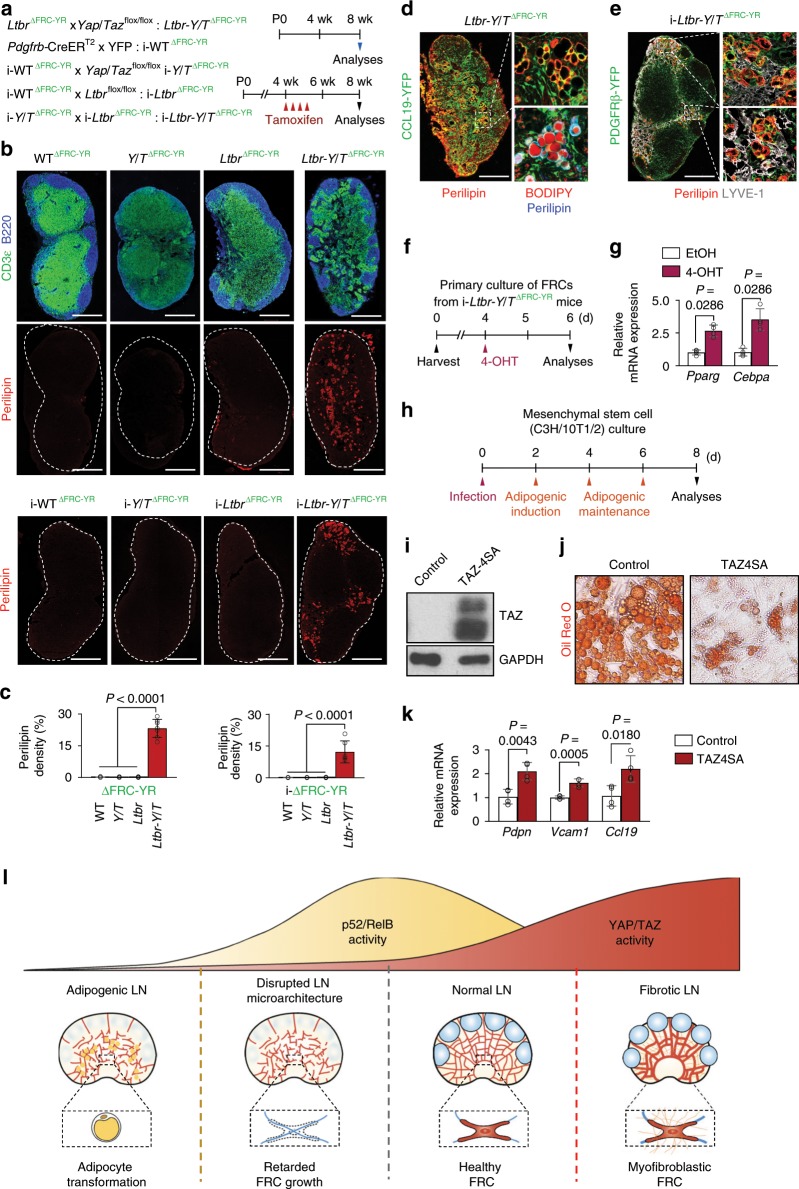
Depletion of Yap/Taz transforms mesenchymal FRC precursors into adipocytes. **a** Diagram for analyses of indicated mice at 8-weeks old with or without tamoxifen delivery from 4-weeks old. **b**, **c** Representative images and comparisons of perilipin^+^ adipocytes within the inguinal LN (dashed line) in indicated mice (*n* = 7). Scale bars, 400 µm. **d** Representative images of inguinal LN filled with adipocytes in *Ltbr-Y*/*T*^∆FRC-YR^ mice (*n* = 6). Right upper panel shows the magnified view of the region within the white dashed box and yellow arrowheads in the right lower panel indicate CCL19-YFP^+^perilipin^+^BODIPY^+^ adipocytes. Scale bars, 500 µm (left panel); 100 µm (right lower panel). **e** Representative images of perilipin^+^ adipocytes along the LYVE-1^+^ lymphatic vessels (dashed-boxes) in inguinal LN of i-*Ltbr-Y*/*T*^∆FRC-YR^ mice (*n* = 6). Scale bar, 400 µm. **f**, Diagram for primary culture of FRCs derived from i-*Ltbr-Y*/*T*^∆FRC-YR^ mice for 4 days and treatment with EtOH or 4-OHT for their analyses at 2 days after the treatment. **g** Comparisons of indicated mRNA expression normalized to *Gapdh* in primary cultured FRCs after treatment with EtOH or 4-OHT for 2 days (*n* = 4). **h** Diagram for adipogenic culture of mesenchymal stem cells (C3H/10T1/2) infected with an adenovirus to induce overexpression of active TAZ (TAZ4SA) for their analyses at 8 days after the infection. **i** Immunoblot analyses of indicated proteins in mesenchymal stem cells (C3H/10T1/2) infected with an adenovirus to induce overexpression of active TAZ (TAZ4SA) or control. **j** Representative images of Oil Red O staining in mesenchymal stem cells (C3H/10T1/2) induced with adipogenic cocktail after infected with an adenovirus to induce overexpression of active TAZ (TAZ4SA). **k** Comparisons of indicated mRNA expression normalized to *Gapdh* in mesenchymal stem cells (C3H/10T1/2) infected with an adenovirus to induce overexpression of active TAZ (TAZ4SA) for their analyses at 2 days after the infection (*n* = 4). **l** Schematic images proposing the importance of coordination of YAP/TAZ activity and LTβR coupling in FRCs during LN growth and maintenance. Unless otherwise denoted, horizontal bars indicate mean ± SD and *P* values versus non-*Y*/*T*^∆FRC-YR^ or non-i-*Ltbr*^∆FRC-YR^ or EtOH or Control by two‐tailed Mann‐Whitney *U* test.

These observations led us to postulate that the binding of YAP/TAZ to p52 is required in myofibroblastic FRC precursors before LTβR engagement. To verify this postulation, we depleted *Yap*/*Taz* in myofibroblastic FRC precursors (*Ltbr*^∆FRC-YR^) by generating *Ltbr-Y*/*T*^∆FRC-YR^ mice, and we also generated i-*Ltbr-Y*/*T*^∆FRC-YR^ mice by crossing *Pdgfrb*-CreER^T2^-YFP reporter mouse (i-WT^∆FRC-YR^) with *Ltbr*^flox/flox^ (i-*Ltbr*^∆FRC-YR^) or *Yap*^flox/flox^/*Taz*^flox/flox^ mouse (i-*Y*/*T*^∆FRC-YR^) (Fig. 6a). Surprisingly, the skin-draining LNs including inguinal, axillary and brachial LNs of *Ltbr-Y*/*T*^∆FRC-YR^ and i-*LTbR-Y*/*T*^∆FRC-YR^ mice had perilipin^+^ adipocytes, which constitutes ~20–25% of the LN density (Fig. 6b, c and Supplementary Fig. 14a).

These aberrant adipocytes were mainly located along the infiltrating lymphatic vessels within the skin-draining LNs, while no adipocytes were observed within the mesenteric LNs of *Ltbr-Y*/*T*^∆FRC-YR^ and i-*Ltbr-Y*/*T*^∆FRC-YR^ mice (Supplementary Fig. 14a). Of note, all the perilipin^+^ or BODIPY^+^ adipocytes were YFP^+^ in LNs of *Ltbr-Y*/*T*^∆FRC-YR^ and i-*Ltbr-Y*/*T*^∆FRC-YR^ mice, indicating that they originated from YFP^+^ FRC precursors (Fig. 6d, e). Further analysis with primary cultured FRCs derived from *Ltbr-Y*/*T*^ΔFRC-YR^ mice showed upregulation of adipogenic genes such as *Pparg* and *Cebpa*^40^ without ectopic fat accumulation (Fig. 6f, g, and Supplementary Fig. 14b), suggesting enhanced adipogenic activity of FRCs rather than metabolic dysregulation in *Ltbr-Y*/*T*^ΔFRC-YR^ mice.

It has been proposed that both adipocytes and LN stromal cells are mesenchymal origin and developmentally related^12^. We therefore examined whether Hippo signaling determines the fate specification of mesenchymal stem cells by utilizing C3H10T1/2 cells (Fig. 6h). Adipogenic culture of C3H10T1/2 cells led to their differentiation into adipocyte-lineage cells, but TAZ4SA overexpressing C3H10T1/2 cells significantly abrogated adipogenesis while they expressed enhanced FRC commitment markers^19,41^ (Fig. 6i–k). These data indicate that YAP/TAZ drive specification and differentiation of mesenchymal cells into FRCs, while they suppress those into other cell types including adipocytes.

## Discussion

FRCs discard some of their myofibroblastic characters as they differentiate and complete maturation, while they gain chemokine secretory functions which are essential for organizing adaptive immunity^12,20^. Here, we show that the canonical Hippo pathway, together with LTβR-non-canonical NF-κB signaling, critically regulates differentiation and maintenance of LN FRCs (Fig. 6l). Deletion of YAP/TAZ in FRCs during development impairs their growth and differentiation, compromising the structural organization of LNs. Hyperactivation of YAP/TAZ in FRCs during the developmental period severely impairs differentiation and maturation of FRCs, leaving non-functional and fibrotic LNs constituted with immature FRCs. Even when YAP/TAZ are hyperactivated in adult mature FRCs, enhanced myofibroblastic characters of FRCs and severe LN fibrosis are similarly observed. These alterations ultimately lead to substantial distortion of LN microarchitecture with impairs adaptive immune responses. Although depletion or hyperactivation of YAP/TAZ leads to similar phenotypes, outcomes of YAP/TAZ depletion are due to loss of FRC pool, while those of YAP/TAZ hyperactivation are owed to maturation defects of FRCs.

These results show that proper modulation of FRCs by canonical Hippo signaling is critical for formation and maintenance of LNs during development and in adult. A previous study proposes that mesenchymal stem cells are differentiated into mature FRCs by suppressing their adipogenicity through promotion of LTβR-p52/RelB signaling^12^. TAZ binding to PPARγ via its WW domain is also demonstrated to inhibit adipogenesis^42^. However, the association between LTβR signaling and YAP/TAZ has not been previously addressed. Our biochemical analyses demonstrate that YAP/TAZ and p52 form a complex to regulate the expression of chemokines such as CCL19 in FRC precursors. Then, upon LTβR activation, YAP/TAZ become phosphorylated and translocated to the cytoplasm, leading to the maturation of FRC precursors. While previous studies demonstrate that LTβR signaling is critical for FRC differentiation, neither ablation of LTβR nor its ligands in FRC precursors is critical for maintaining the FRC lineage^19–21^. Here, we show that depletion of YAP/TAZ in LTβR-ablated FRC precursors induces the transition of the FRC lineage into adipocytes. Collectively, these results indicate that LTβR signaling and YAP/TAZ play both overlapping and independent roles for cell commitment and maintenance of FRCs.

Still, questions remain in linking Yap/Taz modulation in FRCs with its final outcomes. In case of Yap/Taz depletion during development, it results in decreased cellularity of FRCs, which lead to shortage in conduit coverage. Considering that this study agrees with a previous study in that FRC coverage of the LN conduit is not critical for the maintenance of conduit integrity and function^17^, the purpose of FRC coverage around conduits needs further investigation. In contrast, Yap/Taz activation during development lead to maturation defects, and as a consequence impairs LN microarchitecture, which itself could not fully explain the lethal phenotypes. Therefore, the effects of Yap/Taz modulation in other FRC subsets^43,44^ in lymphoid tissues such as spleen and thymus remains to be studied. In addition, exploiting a more specific inducible Cre driver would provide better understanding when comparing the consequences of constitutional or conditional YAP/TAZ manipulation in FRCs. In conclusion, our study clearly demonstrates that the Hippo pathway plays pivotal roles in maturation and maintenance of FRCs of LNs.

## Methods

### Mice

Specific pathogen-free (SPF) C57BL/6J mice (#000664), *Rosa26*-tdTomato mice (#007914), *Rosa26*-eYFP (#007903), *Actb*-DsRed (#006051), *Actb*-GFP (#003291), *Lgr5*-Cre (#008875), and OT-II (#004194) mice were purchased from the Jackson Laboratory. *Lats1*^flox/flox^ ^29^*, Lats2*^flox/flox ^^30^, *Yap*^flox/flox ^^27^*, Taz*^flox/flox ^^28^, *Ltbr*^flox/flox^ ^20^, *Ccl19-*Cre^20^, and *Pdgfrb-*Cre-ER^T2^ ^45^ mice were transferred, established, and bred in SPF animal facilities at KAIST. All mice were maintained in the C57BL/6 background and fed with free access to a standard diet (PMI LabDiet) and water. In order to induce Cre activity in the Cre-ER^T2^ mice, 2 mg of tamoxifen (Sigma-Aldrich) was dissolved in corn oil (Sigma-Aldrich) and intra-peritoneally (i.p.) injected at indicated time points. All mice were anesthetized with i.p. injection of a combination of anesthetics (80 mg/kg ketamine and 12 mg/kg of xylazine) before being euthanatized. We complied with all ethical regulations for animal testing and research and performed all animal experiments and euthanasia under the approval from the Institute Animal Care and Use Committee (No. KA2016-12) of Korea Advanced Institute of Science and Technology (KAIST). Mouse model nomenclatures are included in Supplementary Table 1.

### Histological analyses

Gross images of LNs were acquired using AxioZoom V16 stereo zoom microscope (Carl Zeiss). For LN weight measurement, bilateral inguinal LNs were pooled and weighed using an analytical balance (Mettler Toledo). For immunofluorescence staining of LNs, harvested samples were fixed in 1% paraformaldehyde (PFA) in PBS overnight at 4 °C and dehydrated in 20% sucrose in PBS overnight at 4 °C. Samples were embedded in tissue freezing medium (Leica) and frozen blocks were cut into 20-μm-thick sections. Samples were blocked with 5% goat (or donkey) serum in 0.3% Triton X-100 in PBS (PBST) and incubated for overnight at 4 °C with primary antibodies (diluted at a ratio of 1:200 in blocking solution). After several washes with PBS, samples were incubated for 2 h at RT with secondary antibodies (diluted at a ratio of 1:1000 in blocking solution). After washing several times with PBS, samples were mounted with fluorescent mounting medium (DAKO) and images were acquired using LSM780 or LSM880 confocal microscope (Carl Zeiss).

To examine YAP and TAZ distribution in human LNs, several cervical LNs around thyroid papillary carcinoma were collected from the patients undergoing thyroidectomy with written informed consent according to the protocol approved by the institutional review board of Pusan National University (H-1610-002-003) and Samsung Medical Center (2018-06-061).

For whole-mount staining of embryo, pregnant mice were anesthetized at the indicated day of embryonic development after vaginal plug, and the uteri were removed. Embryos were dissected and the yolk sacs were used for verification of genotype. Inguinal fat pads and LN anlagen were isolated together with the covering skin and processed for staining. Samples were fixed in 2% PFA for 2 h at 4 °C and washed several times with PBS for the staining.

For staining of primary cultured FRCs, cells were plated in 8-well Nunc Lab-Tek II chamber slides (Sigma-Aldrich) and fixed with 4% PFA for 8 min at RT. After several washes with PBS, samples were blocked with 5% goat (or donkey) serum in PBST for 30 min at RT. Cells were incubated with primary antibodies (diluted at a ratio of 1:200 in blocking solution) for overnight at 4 °C. The following primary and secondary antibodies were used in the immunostaining: anti-YAP (rabbit monoclonal, D8H1X, Cell Signaling), anti-TAZ (rabbit polyclonal, HPA007415, Sigma-Aldrich), anti-PDPN (syrian hamster monoclonal, 127402, Biolegend), anti-CCL19 (goat polyclonal, PA5-47958, Thermo Fisher), anti-CCL21 (goat polyclonal, AF457, R&D), anti-ER-TR7 (rat monoclonal, sc-73355, Santa Cruz), anti-CD3e (hamster monoclonal, 145-2C11, BD), anti-B220 (rat monoclonal, RA3-6B2, BD), anti-CD31 (hamster monoclonal, 2H8, Millipore), anti-PDGFRβ (rat monoclonal, APB5, eBioscience), anti-collagen IV (rabbit polyclonal, ab6586, Abcam), anti-Ki-67 (rabbit monoclonal, SP6, Abcam), anti-LYVE-1 (rabbit polyclonal, 11-034, Angiobio), anti-caspase-3 (rabbit polyclonal, 9661, Cell Signaling), anti-vimentin (chicken polyclonal, AB5733, Millipore), anti-collagen I (rabbit polyclonal, ab34710, Abcam), anti-LTβR (rabbit polyclonal, ab70063, Abcam), anti-CD4 (rat monoclonal, GK1.5, BD), anti-CD11b (rat monoclonal, M1/70, BD), anti-CD11c (hamster monoclonal, N418, Bio-Rad), anti-perilipin (guinea pig polyclonal, 20R-PP004, Fitzgerald), anti-PNAd (rat monoclonal, MECA-79, BD), anti-ICAM1 (rat monoclonal, YN1/1.7.4, Abcam), anti-Prox1 (rabbit polyclonal, 102-PA32, ReliaTech), and FITC- or Cy3-conjugated anti-αSMA (mouse monoclonal, 1A4, Sigma-Aldrich). FITC-, Cy3- or Cy5-conjugated secondary antibodies were purchased from Jackson ImmunoResearch. Lipids were stained with BODIPY (Invitrogen) and nuclei were stained with DAPI (Invitrogen).

### Immunological analysis

For adoptive transfer of labeled cells, mononuclear cells were isolated from the spleen and skin-draining LNs from *Actb*-DsRed and *Actb*-GFP mice. B cells from *Actb*-DsRed mice and T cells from *Actb*-GFP mice were further sorted using Dynabeads Untouched Mouse T Cells Kit and Pan B cell Kit (Thermo Fisher). Mixture of 1 × 10^7^ T cells and 1 × 10^7^ B cells were transferred intravenously into the mouse. LNs of the recipient mice were isolated and analyzed at 24 h after the adoptive transfer.

For influenza viral infection, mice were immunized by intranasal administration of 1 × 10^3^ pfu of A/PR/8 [A/Peuerto Rico/8/34 (H1N1)]. Then the mice were monitored for weight loss and survival every day for the next 14 days. For detection of influenza-specific antibodies, ELISA plates (Falcon) coated with 20 μg/ml of formalin-inactivated A/PR/8 virus in PBS were incubated overnight at 4 °C. Blocking with 1% BSA in PBS was performed at 37 °C for 1 h. Serial two-fold dilutions of samples (starting from 1:64 of serum) were applied to plates and incubated for 4 h at 37 °C. Horseradish peroxidase-conjugated goat anti-mouse IgG antibodies (Southern Biotechnology Associates) were added to each well and incubated for 2 h at 37 °C. For color development, TMB-H_2_O_2_ solution (Moss) was added as chromogen substrate to each well and incubated for 15 min at RT. Once stopping solution had been added (0.5 N HCl), absorbance was measured at 450 nm using an ELISA reader (Molecular Devices). Endpoint titers of A/PR/8 virus-specific antibodies were expressed as reciprocal log2 titers of the highest dilution that showed a level of >0.1 absorbance over background.

For intracellular cytokine staining, BD Cytofix/Cytoperm Plus (BD Pharmigen) was used according to the manufacturer’s instructions. For the measurement of IFNγ-producing CD8^+^ T cells, mononuclear cells were obtained from mediastinal LNs and were incubated with 5 ng/ml of PMA and with 500 ng/ml of Ionomycin in the presence of GolgiPlug (BD Pharmingen) for 4 h. The cells were stained with anti-CD8 antibody (rat monoclonal, 53-6.7, BD) and anti-IFN-g antibody (rat monoclonal, XMG1.2, BD), and analyzed with FACS Canto II (BD Biosciences).

For systemic depletion of T cells, anti-mouse CD33 monoclonal Ab (1 mg/kg/day for 5 days; Clone 145-2C11, ATCC) was intravenously administered into WT mice. As a control, the same amount of hamster anti-mouse IgG isotype Ab (R&D Systems) was administered in the same manner.

For analysis of T cell proliferation in vivo, OVA-specific T cells were isolated from OT-II mice, labeled with 10 μM CFSE, and then adoptively transferred intravenously into the mice. Twenty-four hours after the transfer, 100 μg of OVA was injected via footpad. Three days after the injection, mice were euthanatized and mononuclear cells were isolated from popliteal LNs. Activation and CFSE dilution of adaptive transferred OT-II CD4^+^ T cells were analyzed by flow cytometry.

### Electron microscopy

LNs were fixed in 2.5% glutaraldehyde in 0.1 M phosphate buffer (pH 7.4) overnight at 4 °C and post-fixed with 1% osmium tetroxide for 2 h at RT. Samples were dehydrated with series of increasing ethanol concentrations followed by resin embedding. 70-nm-thick ultrathin sections were obtained with an ultramicrotome (UltraCut-UCT, Leica), which were then collected on copper grids. After staining with 2% uranyl acetate and lead citrate, samples were examined by transmission electron microscope (Tecnai G2 Spirit Twin, FEI) at 120 kV.

### LN conduit analysis

Conduit staining was achieved by injecting 10 µL of saturated FITC (Sigma-Aldrich) in HBSS (0.1 mg/mL) into the footpad, and the draining popliteal LN was collected at 10 m after the injection. Samples were fixed in 1% PFA for 1 h immediately after the harvest and processed for whole-mount and imaging.

### Morphometric analyses

Morphometric analyses of the LNs were performed with images taken from mid-sectioned LNs by photographic analysis using ImageJ software (http://rsb.info.nih.gov/ij) or ZEN 2012 software (Carl Zeiss). Numbers of Ki-67^+^ proliferating or caspase-3^+^ apoptotic FRCs, T cells, and B cells were measured from random four or five 0.045 mm^2^ fields of LNs and averaged. Nuclear YAP was counted by calculating the proportion of PDGFRβ^+^ FRCs with nuclear YAP from random four or five 0.045 mm^2^ fields of LNs and averaged. The relative expressions of indicated markers were calculated as PDPN^+^, αSMA^+^, PDGFRβ^+^, collagen IV^+^, CCL19^+^ or CCL21^+^ area divided by Tomato^+^, YFP^+^ or PDGFRβ^+^ area. For density measurements, PDPN^+^, αSMA^+^, PDGFRβ^+^, collagen IV^+^ or ER-TR7^+^ area was measured in random four or five 0.045 mm^2^ area and presented as a percentage of the total measured area. FRC surface area was assessed by employing a 3D reconstruction analysis using Imaris (Bitplane)^46^. The surface area of Tomato^+^ FRCs was calculated using the single-cell morphometric ‘surface’ module in Imaris after the 3D reconstruction of a given image and volume filter was used to reduce background noise. Random four or five 0.045 mm^2^ fields of LNs were analyzed and individual FRC was distinguished as a separate 3D surface object by using the ‘cutting’ tool and DAPI staining was utilized to identify the cell nuclei belonging to each FRC. Percentage of naked conduit and fibril irregularity were measured in up to eight random area of immunofluorescence or TEM images and averaged. Fibril irregularity was assessed by employing a previously described method^47^. Longitudinal section of TEM image was analyzed for non-condensed fibril bundles and unpacked collated fibers with discontinuous, multi-directional pattern were defined as irregular fibrils.

### Flow cytometry and cell sorting

Skin-draining LNs (axillary, brachial, cervical, and inguinal LNs) were harvested and cut into small pieces for digestion^48^. Briefly, LNs were digested in 2 ml of enzyme buffer containing 2 mg/ml collagenase type II (Worthington Biochem), 0.1 mg/ml DNase (Roche), and 1 mg/ml dispase (Gibco) at 37 °C for 30 min. Tissues were gently agitated and pipetted 1–2 times during digestion to disrupt any cell clumps. When LNs were completely digested, cell suspension was filtered through 40 μm nylon cell strainer and washed. Cells were incubated for 20 min with anti-CD45 Microbeads (Miltenyi). To enrich the stromal cell fraction, hematopoietic cells were depleted using AutoMACS (Miltenyi), according to the manufacturer’s instructions. The cells isolated from skin-draining LNs, spleen, and bone marrow were filtered through a 40 μm nylon mesh to remove cell clumps. After RBC lysis by suspension in ACK lysis buffer for 5 min at RT, the cells were incubated for 30 min with antibodies (diluted at a ratio of 1:200) in FACS buffer (5% bovine serum in PBS). After several washes, cells were analyzed by FACS Canto II (BD Biosciences) and the acquired data were further evaluated by using FlowJo software (Treestar). Cell sorting was performed with FACS Aria II (Beckton Dickinson). Dead cells were excluded using DAPI staining (Sigma-Aldrich). The following antibodies were used other than the antibodies described above: anti-CD45 (rat monoclonal, 30-F11, eBioscience), anti-TER-119 (rat monoclonal, TER-119, eBioscience), anti-PDPN (syrian hamster monoclonal, 8.1.1, Biolegend), anti-CD31 (rat monoclonal, MEC 13.3, BD), and anti-CD19 (rat monoclonal, 6D5, Biolegend).

For cell cycle analysis, mice were injected with 1 mg of BrdU solution. Skin‐draining LNs were harvested at 16 h after the injection of BrdU and isolated cells were processed with the APC BrdU flow kit (BD Biosciences) according to the manufacturer’s protocols. The cell cycle profiles were analyzed with a FACS Canto II (BD Biosciences) and assessed with FlowJo (Treestar).

### ATAC sequencing

Approximately 20,000 tdTomato^+^ *Lgr5*^+^ stem cells in dermis were FACS-purified and used for ATAC-seq. The ATAC-seq libraries were prepared by employing a previously described method^49^. 2 × 101 paired-end sequencing was performed on Illumina HiSeq-2500. We randomly sampled 2.5 M reads from each sample using samtools view and pooled them into one file so that each sample is equally represented. Peaks were called on the pooled file as discussed in the previous paragraph. We then determined the number of samples overlapping with each master peak using peaks called on individual samples.

### Quantitative RT-PCR and RNA sequencing

For quantitative RT-PCR, total RNA was extracted from sorted cells by using Trizol RNA extraction kit (Invitrogen) according to the manufacturer’s instructions. Total RNA was reverse transcribed into cDNA using GoScript Reverse Transcription Kit (Promega). Then, quantitative real-time PCR was performed using FastStart SYBR Green Master mix (Roche) and Bio-rad S1000 Thermocycler. GAPDH was used as a reference gene and the results were presented as relative expressions to control. List of primer sequences are described in Supplementary Table 2.

For RNA sequencing, the construction of library was performed using QuantSeq 3′ mRNA-Seq Kit (Lexogen GmbH, Austria) according to the manufacturer’s instructions. In brief, each 500 ng total RNA were prepared and an oligo-dT primer containing an Illumina-compatible sequence at its 5′ end was hybridized to the RNA and reverse transcription was performed. After degradation of the RNA template, second strand synthesis was initiated by a random primer containing an Illumina-compatible linker sequence at its 5′ end. The double-stranded library was purified by using magnetic beads to remove all reaction components. The library was amplified to add the complete adapter sequences required for cluster generation. The finished library was purified from PCR components. High-throughput sequencing was performed as single-end 75 sequencing using NextSeq 500 (Illumina). Mapping of RNA-Seq reads were performed using Bowtie2 version 2.1.0, which was used to bring together transcripts, assess their exuberances, and identify DEGs or isoforms using cufflinks. The RT (Read Count) data were processed based on Quantile normalization method using the Genowiz version 4.0.5.6 (Ocimum Biosolutions). The Ingenuity Pathway Analysis tool (QIAGEN) was used to interpret data in the context of canonical pathways, biological processes and networks. Both up- and downregulated identifiers were defined as value parameters for the analysis. Significance of the canonical pathways and biological function and networks were tested by the Fisher Exact test *p*-value, and determined for their activation or inhibition upon activation z-score. For GSEA, gene sets from the Molecular Signatures Database (MSigDB) 5.2 were used for the analysis of the Hippo pathway-responsive transcriptomes. Cluster analysis and heatmap construction were performed using Multiple Experiment Viewer (MeV) from The Institute of Genomic Research (TIGR) and Cluster and TreeView from the Eisen laboratory. Original data are available in the National Center for Biotechnology Information’s Gene Expression Omnibus (accession number GSE89742). For presentation of RNA-sequencing data into bar graphs, normalized raw counts (log2) were converted by using the following formula: 2^(x: normalized raw count)^. Next, given values were divided by the average value of WT and presented as relative ratio.

### Primary culture of FRCs

Skin-draining lymph nodes of indicated mice were harvested, cut into small pieces, and digested^48^. LNs were digested in 2 ml of enzyme buffer containing mixture of 2 mg/ml collagenase type II (Worthington Biochem), 0.1 mg/ml DNase (Roche), and 1 mg/ml dispase (Gibco) at 37 °C for 30 min. Tissues were gently agitated and pipetted several times during digestion to disrupt any cell clumps. When LNs were completely digested, cell suspension was filtered through 40 μm nylon cell strainer and washed. Cells were incubated for 20 min with anti-CD45 Microbeads (Miltenyi). To enrich the stromal cell fraction, hematopoietic cells were depleted using AutoMACS (Miltenyi), according to the manufacturer’s instructions. In order to induce FRC differentiation, cells were incubated with DMEM containing 10% fetal bovine serum and 500 ng/ml of LTβR agonistic antibody (Abcam, Cat. ab65089) for 5 days. For induction of cre activity in primary cultured FRCs derived from *Lats1/2*^i∆FRC^ or *Yap/Taz*^∆FRC^ mice, cells were treated with 5 µM 4-hydroxy-tamoxifen (4-OHT) in 100% ethanol (EtOH) or 100% EtOH alone for 2 days as a control. For primary culture of human FRCs, cervical LN specimens were acquired for diagnostic purposes from presently healthy adults with written informed consent according to the protocol approved by the institutional review board of Samsung Medical Center (2018-06-061). LNs were cut into small pieces immediately after the harvest and digested^50^. Following the digestion, cells were covered with media on a tissue culture plate and grown for 4 days to permit fibroblasts to emerge. Culture-expanded monolayer of FRCs under passage 3 that were validated as CD45^−^, CD31^−^, and PDPN^+^ were used for the experiments.

For adenoviral infection, human TAZ4SA and YAP5SA cDNAs^51^ were cloned into the pAdtrack-CMV-GFP vector^52^. Cloning vectors were then recombined with the pAdEasy-1 vector in BJ5183-AD-1 electroporation–competent cells (Agilent Technologies). The recombinant DNA was linearized with PacI and introduced into 293AD cells by transfection with Lipofectamine LTX and PLUS reagent (Invitrogen). After verification of GFP expression, cell pellets were centrifuged and resuspended with 10% glycerol in PBS and lysed with 4 freeze-thaw cycles. Adenoviruses were purified by ultracentrifugation at 46,000 × *g* for 2 h at 4 °C within a discontinuous gradient from 2.2 to 4.0 M CsCl (Amresco) in 10 mM HEPES (Sigma-Aldrich). The adenovirus-containing layer was removed with a syringe needle, and the viruses were washed twice in a solution containing 10 mM Tris-HCl (pH 8.0) and 2 mM MgCl_2_ using an Amicon Ultra Centrifugal Filter (Sigma-Aldrich). Virus titration was performed by counting exposed 293AD or target cells positive for GFP with a fluorescence microscope.

### Immunoblotting

For immunoblot analysis, cells were lysed on ice in RIPA lysis buffer supplemented with protease and phosphatase inhibitors (Roche). Cell lysates were centrifuged for 10 min at 4 °C, 13,000 rpm. Protein concentrations of the supernatants were quantitated using the detergent-insensitive Pierce BCA protein assay kit (Thermo Fischer, 23227). Laemmli’s buffer was added to total protein lysates and samples were denatured at 95 °C for 5 min. Aliquots of each protein lysate (10–20 μg) were subjected to SDS polyacrylamide gel electrophoresis. After electrophoresis, proteins were transferred to nitrocellulose membranes and blocked for 30 min with 5% skim milk in TBST (0.1% Tween 20 in TBS). For phosphorylated protein detection, membranes were blocked with 2% BSA in TBS. For detection of protein stability, protein synthesis was blocked by treatment of 50 μg/ml cycloheximide (CHX) for indicated time. Primary antibodies (diluted at a ratio of 1:1000 in blocking solution) were incubated overnight at 4 °C. After washes, membranes were incubated with anti-rabbit (CST, #7074) or anti-mouse (CST, #7076) secondary peroxidase coupled antibody (diluted at a ratio of 1:5000 in TBST) for 1 h at RT. Target proteins were detected using ECL western blot detection solution (Millipore, WBKLS0500). The following antibodies were used for immunoblotting: anti-YAP (rabbit monoclonal, D8H1X, Cell Signaling), anti-phospho-YAP (rabbit polyclonal, 4911, Cell Signaling), anti-YAP/TAZ (rabbit monoclonal, D24E4, Cell Signaling), anti-TAZ (rabbit monoclonal, V386, Cell Signaling), anti-LATS1 (rabbit monoclonal, C66B5, Cell Signaling), anti-phospho-LATS1/2 (rabbit monoclonal, D57D3, Cell Signaling), anti-CTGF (rabbit polyclonal, ab6992, Abcam), anti-RelB (rabbit monoclonal, D7D7W, Cell Signaling), anti-CTGF (rabbit polyclonal, ab6992, Abcam), anti-p100/p52 (rabbit polyclonal, 4882, Cell Signaling), anti-p100/p52 (rabbit monoclonal, sc-7386, Santa Cruz), anti-NIK (rabbit polyclonal, 4994, Cell Signaling), anti-LaminB (rabbit monoclonal, D4Q4Z, Cell Signaling), anti-GAPDH (rabbit monoclonal, D16H11, Cell Signaling), and anti-β actin (rabbit monoclonal, AC-74, Sigma-Aldrich).

For nuclear‐cytoplasmic fractionation of cells, harvested cells were resuspended in hypotonic lysis buffer (10 mM HEPES [pH 7.8], 10 mM KCl, 1.5 mM MgCl_2_, 0.5 mM DTT, and protease inhibitors). To break plasma membrane, the resuspended cells were mixed with 0.3% NP-40 by vortexing for 5 s, and the cytoplasmic fraction was obtained from the supernatant after centrifugation. After several washes with PBS, the pellet was boiled in Laemmli sample buffer and used as the nuclear fraction. The uncropped and unprocessed scans with marker positions of all blots were included in the Source Data file.

### Pull-down assay and co-immunoprecipitation

The N-terminal Flag-SBP tagged human TAZ4SA cDNAs were cloned into the pcDNA3.1 vector (Thermo Fischer, Cat. V790-20). Flag-p52 and Flag-RelB were purchased from Addgene (plasmid # 20019, # 20017). Each mutant construct (TAZ4SA△W, p52 YA and RelB YA) was generated by overlap extension PCR. HEK293T cells were cultured in 6‐well plate and were co-transfected with indicated constructs using polyethylenimine Max (Polyscience, Cat. 24765-1). Two days after transfection, the cells were harvested, lysed with NETN buffer (20 mM Tris-HCl (pH 7.4), 100 mM NaCl, 1 mM EDTA, 0.5% Nonidet P-40, and protease inhibitors). Cell extracts (1 mg) were incubated with Streptavidin Agarose (Pierce, Cat. 20359) for 2 h at 4 °C, and then the beads were washed three times with lysis buffer and boiled with Laemmli sample buffer. For co-immunoprecipitation, harvested FRCs were lysed with NETN buffer. Cell extracts (0.5 mg) were incubated overnight at 4 °C with 0.5 μg of anti-IgG (rabbit polyclonal, H-270, Santa Cruz) or anti-YAP/TAZ (rabbit monoclonal, D24E4, Cell Signaling) antibody. The extracts were incubated with 20 μl of protein A/G agarose beads (Pierce) for 1 h, and then the beads were washed three times with lysis buffer (Triton X‐100 reduced to 0.1%) and boiled with Laemmli sample buffer. For detection of p52, the TrueBlot anti-Rabbit IgG HRP (ROCKLAND, Cat. 18-8816-31) was used as secondary antibody for reducing the heavy chain interference.

### Luciferase assay

The indicated portion of the *Ccl19* genomic locus including p52 binding site was cloned into the pGL3-Basic vector (Promega, Cat. E1751). Mutant construct was generated with deletion of p52 binding site by overlap extension PCR. HEK293T cells were cultured in 24‐well plate and were co-transfected with a 100 ng HA‐TAZ4SA (WT or △W), 100 ng Flag‐p52 (WT or YA), 200 ng CCL19 luciferase (WT or Mut) or 20 ng CMV‐Renilla per well by using polyethylenimine Max. Twenty-four hours later, cells were harvested, lysed, and assayed with the Dual Luciferase Reporter Assay System (Promega, Cat. E1960). List of primer sequences for luciferase assay are described in Supplementary Table 2.

### ChIP-qPCR analysis

MEF cells were fixed with 1% formaldehyde for 10 min and then neutralized with 125 mM glycine for 5 min at RT. The cells were washed with PBS and then lysed with ChIP dilution buffer [50 mM HEPES (pH 7.5), 155 mM NaCl, 1% Triton X-100, 0.1% sodium deoxycholate, 123 mM EDTA] containing 1% SDS. The DNA in the cell lysates was fragmented by sonication using a bioruptor sonicator. The cell lysates were centrifuged at 13,000 rpm for 15 min at 4 °C, and the resulting supernatants were further diluted with ChIP dilution buffer. They were then incubated overnight at 4 °C with either TAZ antibody (Sigma, HPA007415) or IgG (Santa Cruz Biotechnology). The next day, protein A/G beads (Gendepot) were added and the samples were incubated for an additional 3 h at 4 °C. The beads were then isolated with centrifugation, washed with ChIP wash buffer [10 mM Tris-HCl (pH 8.0), 250 mM LiCl, 0.5% Nonidet P-40, 0.5% sodium deoxycholate, 1 mM EDTA], and suspended in SDS lysis buffer [50 mM Tris-HCl (pH 8.0), 10 mM EDTA, 1% SDS] for overnight incubation at 65 °C. The beads were then removed, and the remaining material was incubated for 2 h at 55 °C with proteinase K (20 mg/ml) and glycogen (20 mg/ml). After a final 1 h incubation with RNaseA at 37 °C, the DNA was purified using standard procedures and analyzed for “GGGRNNYYCC” motif. List of primer sequences for ChIP-qPCR are described in Supplementary Table 2.

### In situ proximity ligation assay

Cells cultured on confocal dishes were fixed with 4% paraformaldehyde for 20 min at RT, permeabilized and incubated with primary antibodies at 4 °C. For in situ proximity ligation assay, protein–protein interactions between YAP or TAZ and p52 were detected with secondary proximity probes (Anti-Rabbit Plus and Anti-Mouse Minus) according to the Duolink in situ Fluorescence User Guide (Sigma-Aldrich).

### Adipogenic induction

In order to induce adipogenic differentiation, confluent cells were incubated with adipogenic differentiation medium [DMEM containing 10% fetal bovine serum, 5 μg/ml insulin (Sigma-Aldrich), 0.5 mM 3-isobutyl-1-methylxanthine (IBMX, Sigma-Aldrich) and 1 μM dexamethasone (Sigma-Aldrich)]. After 3 days, adipogenic differentiation medium was replaced with maintenance medium (DMEM containing 10% fetal bovine serum and 5 μg/ml insulin). For Oil Red O staining, cells were fixed with 4% paraformaldehyde for 40 min at RT. After being washed with PBS and 60% isopropanol, cells were incubated with filtered Oil Red O working solution for 50 min at RT. After staining, several washes with PBS and 60% isopropanol were performed to reduce non-specific staining. Images of stained cells were captured by a microscope equipped with a CCD camera (Carl Zeiss).

### Statistical analyses

All values are presented as mean ± standard deviation (SD). For continuous data, statistical significance was determined by the Mann–Whitney *U* test or Student’s *t*-test between two groups and one-way ANOVA for multiple-group comparison. The survival curve was evaluated using the Kaplan–Meier method, and the statistical difference was analyzed using the log-rank test. Statistical analysis was performed with GraphPad Prism. Statistical significance was set to *P* < 0.05.

### Reporting summary

Further information on research design is available in the Nature Research Reporting Summary linked to this article.

## Supplementary information

Supplementary Information

Reporting Summary

## Data Availability

The RNA-sequencing and ATAC-sequencing data generated with this study have been deposited in Gene Expression Omnibus under the accession number GSE89742. The source data underlying all Figs. and Supplementary Figs. are provided as a Source Data file. A Reporting summary for this article is available as a Supplementary Information file. All other data that support the findings of this study are available from the corresponding author upon reasonable request.

## Source data

Source Data

## References

[1] Fletcher AL, Acton SE, Knoblich K (2015). Lymph node fibroblastic reticular cells in health and disease. Nat. Rev. Immunol..

[2] Petrova TV, Koh GY (2018). Organ-specific lymphatic vasculature: from development to pathophysiology. J. Exp. Med..

[3] Chang JE, Turley SJ (2015). Stromal infrastructure of the lymph node and coordination of immunity. Trends Immunol..

[4] Bajenoff M, Glaichenhaus N, Germain RN (2008). Fibroblastic reticular cells guide T lymphocyte entry into and migration within the splenic T cell zone. J. Immunol..

[5] Siegert S, Luther SA (2012). Positive and negative regulation of T cell responses by fibroblastic reticular cells within paracortical regions of lymph nodes. Front Immunol..

[6] Cremasco V (2014). B cell homeostasis and follicle confines are governed by fibroblastic reticular cells. Nat. Immunol..

[7] Riedel A, Shorthouse D, Haas L, Hall BA, Shields J (2016). Tumor-induced stromal reprogramming drives lymph node transformation. Nat. Immunol..

[8] Tamura N (2009). Tumor histology in lymph vessels and lymph nodes for the accurate prediction of outcome among breast cancer patients treated with neoadjuvant chemotherapy. Cancer Sci..

[9] Estes JD (2013). Pathobiology of HIV/SIV-associated changes in secondary lymphoid tissues. Immunol. Rev..

[10] Zeng M (2011). Cumulative mechanisms of lymphoid tissue fibrosis and T cell depletion in HIV-1 and SIV infections. J. Clin. Invest..

[11] Lu TT, Browning JL (2014). Role of the lymphotoxin/LIGHT system in the development and maintenance of reticular networks and vasculature in lymphoid tissues. Front Immunol..

[12] Benezech C (2012). Lymphotoxin-beta receptor signaling through NF-kappaB2-RelB pathway reprograms adipocyte precursors as lymph node stromal cells. Immunity.

[13] Buckley CD, Barone F, Nayar S, Benezech C, Caamano J (2015). Stromal cells in chronic inflammation and tertiary lymphoid organ formation. Annu Rev. Immunol..

[14] Barone F (2016). Stromal fibroblasts in tertiary lymphoid structures: a novel target in chronic inflammation. Front Immunol..

[15] Majumder S (2019). IL-17 metabolically reprograms activated fibroblastic reticular cells for proliferation and survival. Nat. Immunol..

[16] van de Pavert SA (2009). Chemokine CXCL13 is essential for lymph node initiation and is induced by retinoic acid and neuronal stimulation. Nat. Immunol..

[17] Kong YY (1999). OPGL is a key regulator of osteoclastogenesis, lymphocyte development and lymph-node organogenesis. Nature.

[18] Kim D (2000). Regulation of peripheral lymph node genesis by the tumor necrosis factor family member TRANCE. J. Exp. Med..

[19] Benezech C (2010). Ontogeny of stromal organizer cells during lymph node development. J. Immunol..

[20] Chai Q (2013). Maturation of lymph node fibroblastic reticular cells from myofibroblastic precursors is critical for antiviral immunity. Immunity.

[21] Onder L (2017). Lymphatic endothelial cells control initiation of lymph node organogenesis. Immunity.

[22] Yu FX, Zhao B, Guan KL (2015). Hippo pathway in organ size control, tissue homeostasis, and cancer. Cell.

[23] Halder G, Dupont S, Piccolo S (2012). Transduction of mechanical and cytoskeletal cues by YAP and TAZ. Nat. Rev. Mol. Cell Biol..

[24] Irvine, K. D. & Harvey, K. F. Control of organ growth by patterning and hippo signaling in Drosophila. *Cold Spring Harb. Perspect. Biol*. **7**, a019224 (2015). 10.1101/cshperspect.a019224 PMC4448604 26032720

[25] Piccolo S, Dupont S, Cordenonsi M (2014). The biology of YAP/TAZ: hippo signaling and beyond. Physiol. Rev..

[26] Cho, H. et al. YAP and TAZ negatively regulate Prox1 during developmental and pathologic lymphangiogenesis. *Circ. Res.* **124**, 225–242 (2019). 10.1161/CIRCRESAHA.118.313707 30582452

[27] Xin M (2011). Regulation of insulin-like growth factor signaling by Yap governs cardiomyocyte proliferation and embryonic heart size. Sci. Signal.

[28] Xin M (2013). Hippo pathway effector Yap promotes cardiac regeneration. Proc. Natl Acad. Sci. USA.

[29] Heallen T (2011). Hippo pathway inhibits Wnt signaling to restrain cardiomyocyte proliferation and heart size. Science.

[30] Kim M (2013). cAMP/PKA signalling reinforces the LATS-YAP pathway to fully suppress YAP in response to actin cytoskeletal changes. EMBO J..

[31] Hiruma K, Hirsch R, Patchen M, Bluestone JA, Gress RE (1992). Effects of anti-CD3 monoclonal antibody on engraftment of T-cell-depleted bone marrow allografts in mice: host T-cell suppression, growth factors, and space. Blood.

[32] Loubaki L, Tremblay T, Bazin R (2013). In vivo depletion of leukocytes and platelets following injection of T cell-specific antibodies into mice. J. Immunol. Methods.

[33] Xiao Y (2018). Hippo signaling plays an essential role in cell state transitions during cardiac fibroblast development. Dev. Cell.

[34] dos Santos G (2015). Vimentin regulates activation of the NLRP3 inflammasome. Nat. Commun..

[35] Cildir G, Low KC, Tergaonkar V (2016). Noncanonical NF-kappaB signaling in health and disease. Trends Mol. Med..

[36] Vallabhapurapu S, Karin M (2009). Regulation and function of NF-kappaB transcription factors in the immune system. Annu Rev. Immunol..

[37] Varelas X (2014). The Hippo pathway effectors TAZ and YAP in development, homeostasis and disease. Development.

[38] Barrallo-Gimeno A, Nieto MA (2009). Evolutionary history of the Snail/Scratch superfamily. Trends Genet..

[39] Tang Y, Feinberg T, Keller ET, Li XY, Weiss SJ (2016). Snail/Slug binding interactions with YAP/TAZ control skeletal stem cell self-renewal and differentiation. Nat. Cell Biol..

[40] Cristancho AG, Lazar MA (2011). Forming functional fat: a growing understanding of adipocyte differentiation. Nat. Rev. Mol. Cell Biol..

[41] White A (2007). Lymphotoxin a-dependent and -independent signals regulate stromal organizer cell homeostasis during lymph node organogenesis. Blood.

[42] Hong JH (2005). TAZ, a transcriptional modulator of mesenchymal stem cell differentiation. Science.

[43] Cheng HW (2019). Origin and differentiation trajectories of fibroblastic reticular cells in the splenic white pulp. Nat. Commun..

[44] Youn DY (2008). Bis deficiency results in early lethality with metabolic deterioration and involution of spleen and thymus. Am. J. Physiol. Endocrinol. Metab..

[45] Sheikh AQ, Misra A, Rosas IO, Adams RH, Greif DM (2015). Smooth muscle cell progenitors are primed to muscularize in pulmonary hypertension. Sci. Transl. Med..

[46] Novkovic M (2016). Topological small-world organization of the fibroblastic reticular cell network determines lymph node functionality. PLoS Biol..

[47] Drumea-Mirancea M (2006). Characterization of a conduit system containing laminin-5 in the human thymus: a potential transport system for small molecules. J. Cell Sci..

[48] Fletcher AL (2010). Lymph node fibroblastic reticular cells directly present peripheral tissue antigen under steady-state and inflammatory conditions. J. Exp. Med..

[49] Buenrostro JD, Giresi PG, Zaba LC, Chang HY, Greenleaf WJ (2013). Transposition of native chromatin for fast and sensitive epigenomic profiling of open chromatin, DNA-binding proteins and nucleosome position. Nat. Methods.

[50] Knoblich K (2018). The human lymph node microenvironment unilaterally regulates T-cell activation and differentiation. PLoS Biol..

[51] Jeong SH (2018). Hippo-mediated suppression of IRS2/AKT signaling prevents hepatic steatosis and liver cancer. J. Clin. Investig..

[52] He TC (1998). A simplified system for generating recombinant adenoviruses. Proc. Natl Acad. Sci. USA.

