# Supplementary material for: YAP/TAZ direct commitment and maturation of lymph node fibroblastic reticular cells
Source: Nat Commun. 2020 Jan 24;11:519. doi: 10.1038/s41467-020-14293-1 (PMC6981200; doi:10.1038/s41467-020-14293-1)
Supplement: Supplementary file 1 — Supplementary Information [file 41467_2020_14293_MOESM1_ESM.pdf]

Supplementary Information

YAP/TAZ direct commitment and maturation of lymph node fibroblastic reticular cells

Choi *et al.*

It includes;

1. Supplementary Figures 1-14
2. Supplementary Tables 1-2

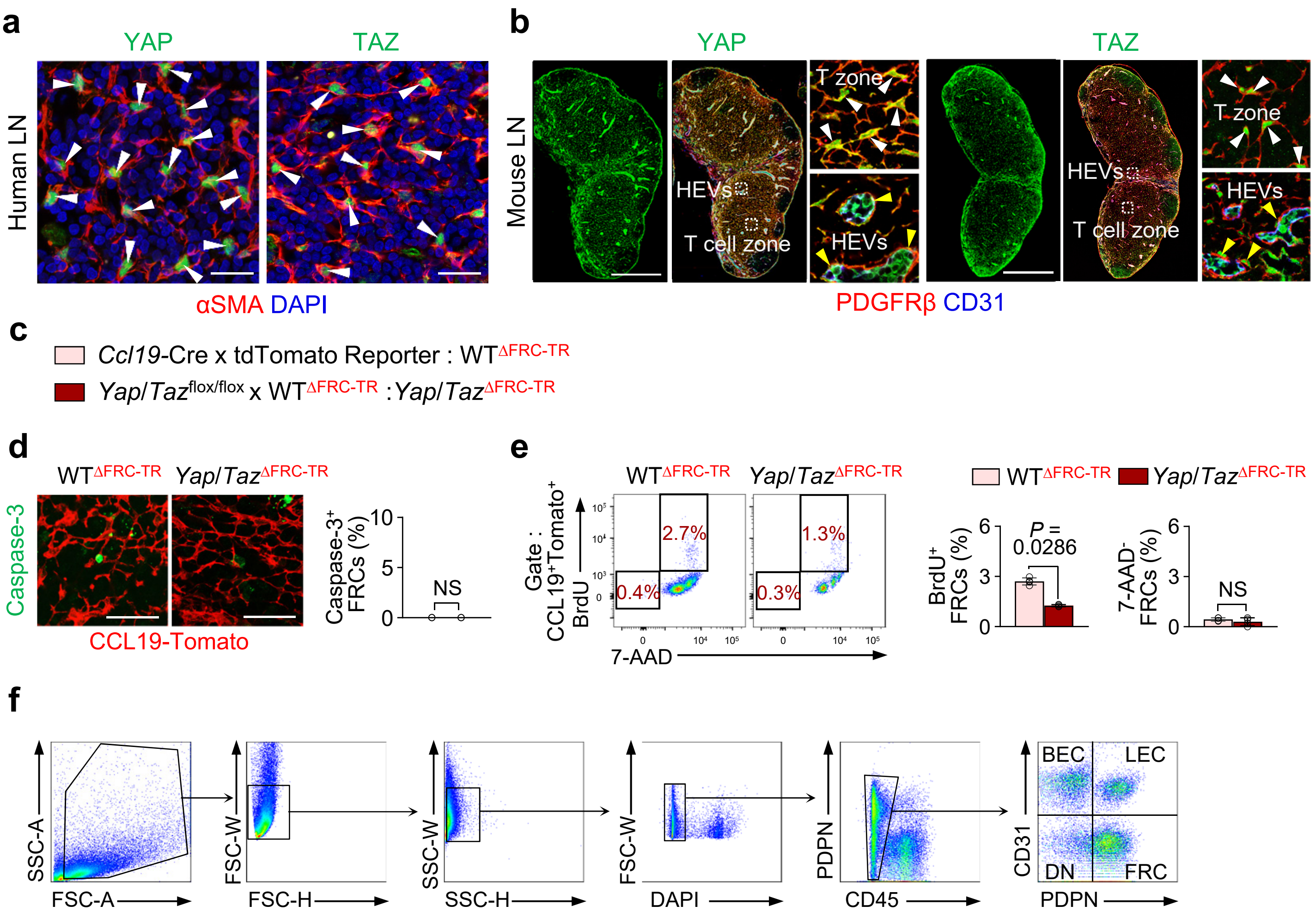

Supplementary Figure 1: YAP/TAZ are enriched in human and murine FRCs and support growth of FRCs.
a, Representative images showing abundant YAP and TAZ expressions (white arrowheads) in αSMA^+ FRCs around T cell zone of presently healthy human cervical LN sample. Nuclei are stained with DAPI. Scale bar, 20 μm. Similar findings were observed in *n* = 6 cervical LNs from three presently healthy patients.
b, Representative images demonstrating high expressions of YAP and TAZ in $PDGFR\beta^+$ FRCs of inguinal LN in 8-weeks-old adult WT mice. FRCs around T cell zone (white arrowheads) and high endothelial venules (HEVs, yellow arrowheads) are magnified as indicated. Scale bars, 500 μm. Similar findings were observed in *n* = 6 mice from three independent experiments.
c, Diagram for generation of indicated mice and their analyses at 8 weeks after birth.
d, Representative images and comparisons of caspase-3^+ apoptotic FRCs of inguinal LN in $WT^{\Delta FRC\text{-}TR}$ and *Yap*/*Taz*$^{\Delta FRC\text{-}TR}$ mice. Scale bars, 50 μm. Each dot indicates a mean value obtained from one mouse and *n* = 4 mice/group pooled from two independent experiments. Horizontal bars indicate mean ± SD and *P* value versus $WT^{\Delta FRC\text{-}TR}$ by two-tailed Mann-Whitney *U* test. NS, not significant.
e, Representative flow cytometric plots and comparison of $BrdU^+$ proliferative or 7-AAD^- apoptotic FRCs gated from $Tomato^+$ cells of skin-draining LNs in $WT^{\Delta FRC\text{-}TR}$ and *Yap*/*Taz*$^{\Delta FRC\text{-}TR}$ mice. Each dot indicates a mean value obtained from one mouse and *n* = 4 mice/group pooled from two independent experiments. Horizontal bars indicate mean ± SD and *P* values versus $WT^{\Delta FRC\text{-}TR}$ by two-tailed Mann-Whitney *U* test. NS, not significant.
f, Gating strategy to sort FRCs ($CD45^-PDPN^+CD31^-$), LECs ($CD45^-PDPN^+CD31^+$), BECs ($CD45^-PDPN^-CD31^+$) or DN cells ($CD45^-PDPN^-CD31^-$) from skin-draining LNs in WT mice.

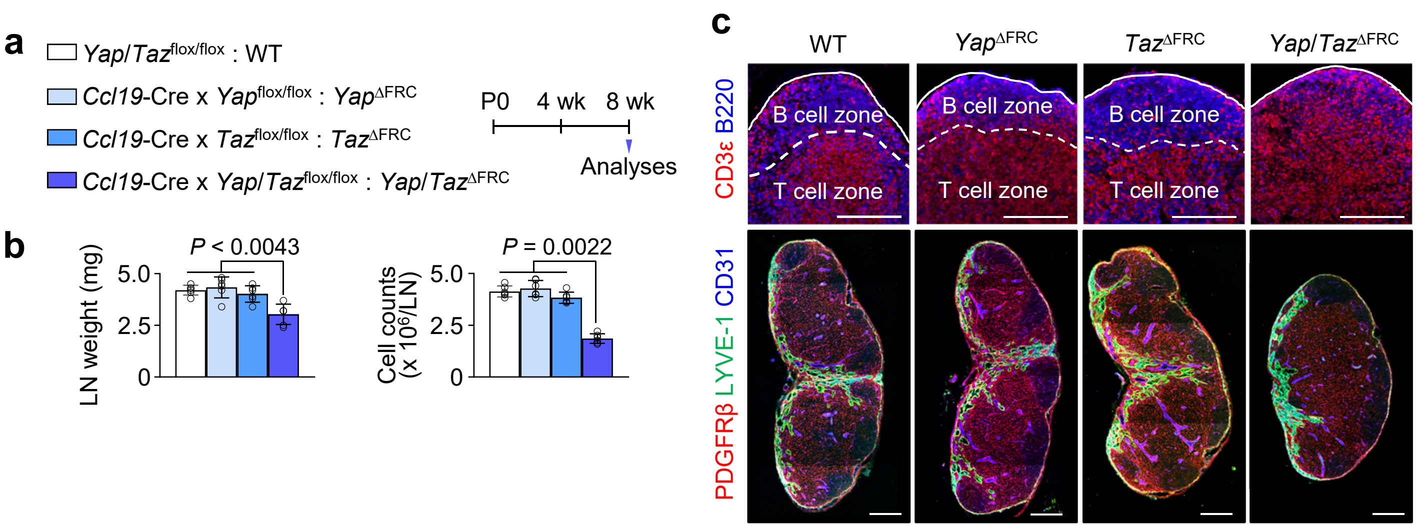

Supplementary Figure 2: Redundant roles of YAP or TAZ in FRCs.

a, Diagram for generation of indicated mice and their analyses at 8-weeks-old.

b, Comparisons of the inguinal LN weight and cellularity within the inguinal LN in WT, *Yap*$^{\Delta FRC}$, *Taz*$^{\Delta FRC}$, and *Yap*/*Taz*$^{\Delta FRC}$ mice. Each dot indicates a mean value obtained from one mouse and n = 6 mice/group pooled from two independent experiments. Horizontal bars indicate mean ± SD and *P* values versus WT, *Yap*$^{\Delta FRC}$, or *Taz*$^{\Delta FRC}$ by two-tailed Mann-Whitney *U* test.

c, Representative images of border between B and T cell zones (upper panels, white dashed line) beneath the LN capsule (white line) and LYVE-1^{+} lymphatic vessels and CD31^{+} blood vessels (lower panels) in indicted mice. Scale bars, 250 μm. Similar findings were observed in *n* = 6 mice/group from two independent experiments.

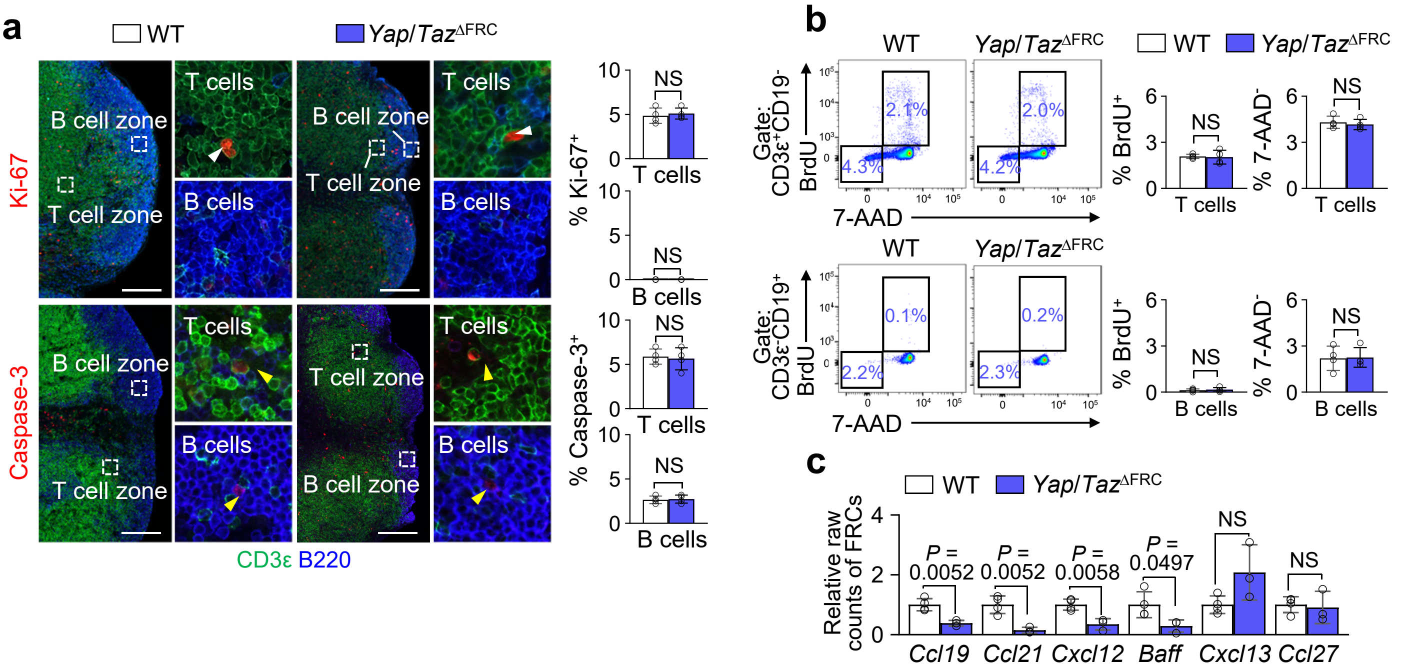

Supplementary Figure 3: Impaired immune cell trafficking in *Yap/Taz*$^{\Delta FRC}$ mice.

a, Representative images and comparisons of Ki-67$^+$ proliferative (white arrowheads) or caspase-3$^+$ apoptotic (yellow arrowheads) T and B cells of the inguinal LN in WT and *Yap/Taz*$^{\Delta FRC}$ mice. The regions within the white dashed box are magnified as indicated. Scale bars, 200 μm. Each dot indicates a mean value obtained from one mouse and n = 4 mice/group pooled from two independent experiments. Horizontal bars indicate mean ± SD. NS, not significant.

b, Representative flow cytometric plots and comparison of BrdU$^+$ proliferative or 7-AAD$^-$ apoptotic T cells (gated from CD3ε$^+$CD19$^-$ cells) and B cells (gated from CD3ε$^-$CD19$^+$ cells) of skin-draining LNs in WT and *Yap/Taz*$^{\Delta FRC}$ mice. Each dot indicates a mean value obtained from one mouse and n = 4 mice/group pooled from two independent experiments. Horizontal bars indicate mean ± SD. NS, not significant.

c, Comparison of relative raw counts of indicated genes from RNA-sequencing data of sorted FRCs from WT and *Yap/Taz*$^{\Delta FRC}$ mice. Each dot indicates a mean of triplicate or quadruplicate experiments using n = 4 (WT) or n = 3 (*Yap/Taz*$^{\Delta FRC}$) mice. Horizontal bars indicate mean ± SD and P values versus WT by two-tailed Student's t-test. NS, not significant.

Supplementary Figure 4

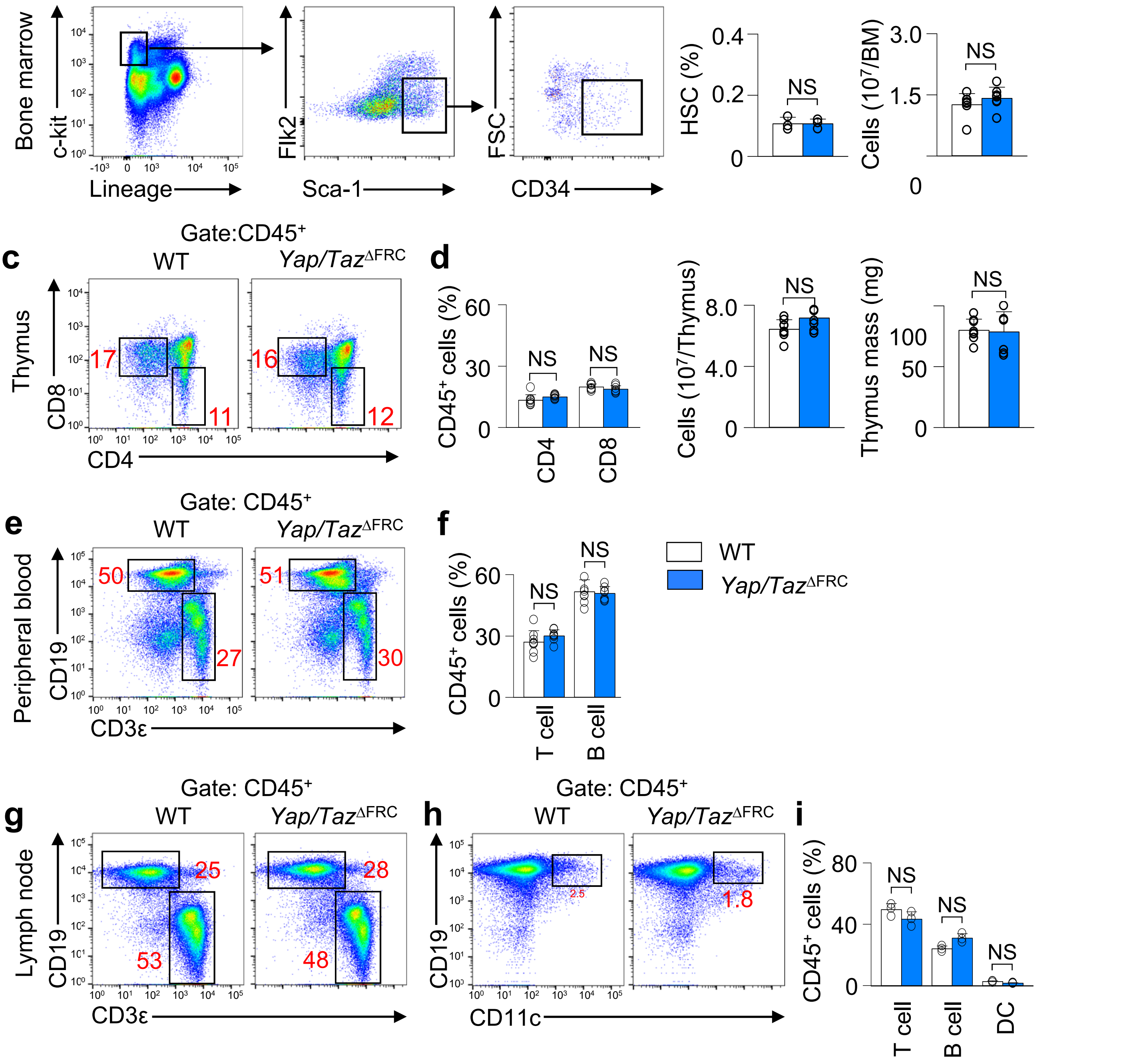

Supplementary Figure 4: No apparent differences in immune cell composition in *Yap/Taz*$^{\Delta FRC}$ mice.
BM, Thymus, LNs, and peripheral blood of 8-week-old WT and *Yap/Taz*$^{\Delta FRC}$ mice were sampled and analyzed.
a,**b**, Flow cytometric plots gated for c-Kit^{+}Flk2^{-}Sca1^{+}CD34^{+} HSC and comparisons of percentage of HSC and cellularity of BM. Each dot indicates a value obtained from one mouse and *n* = 3 (HSC) or *n* = 8 (WT;BM) and 7 (*Yap/Taz*$^{\Delta FRC}$;BM) mice from two independent experiments. Horizontal bars indicate mean ± SD and *P* values versus WT by two-tailed Mann-Whitney *U* test. NS, not significant.
c,**d**, Flow cytometric plots and comparisons of percentages of T cells which are pre-gated as CD45^{+}CD4^{+} or CD8^{+} cells in thymus. Comparisons of total number of cells (10^{7}/thymus) and mass (mg) of thymus. Each dot indicates a value obtained from one mouse and n = 8 (WT) or n = 7 (*Yap/Taz*$^{\Delta FRC}$) mice from two independent experiments. Horizontal bars indicate mean ± SD and P values versus WT by two-tailed Mann-Whitney U test. NS, not significant.
e,**f**, Flow cytometric plots and comparisons of percentages of T and B lymphocytes and monocytes which are pre-gated as CD45^{+} cells in peripheral blood. Numbers indicate percentages of CD19^{-}CD3ε^{+} T cells, CD19^{+}CD3ε^{-} B cells, and CD19^{-}CD11b^{+} monocytes. Each dot indicates a value obtained from one mouse and *n* = 8 (WT) or *n* = 7 (*Yap/Taz*$^{\Delta FRC}$) mice from two independent experiments. Horizontal bars indicate mean ± SD and *P* values versus WT by two-tailed Mann-Whitney *U* test. NS, not significant.
g-**i**, Flow cytometric plots and comparisons of T and B lymphocytes and DCs which are pre-gated as CD45^{+} cells in LNs. Numbers indicate percentages of CD19^{-}CD3ε^{+} T cells, CD19^{+}CD3ε^{-} B cells, and CD19^{-}CD11c^{+} DCs. Dot indicates values obtained from *n* = 3 mice/group. Horizontal bars indicate mean ± SD and *P* values versus WT by two-tailed Mann-Whitney *U* test. NS, not significant.

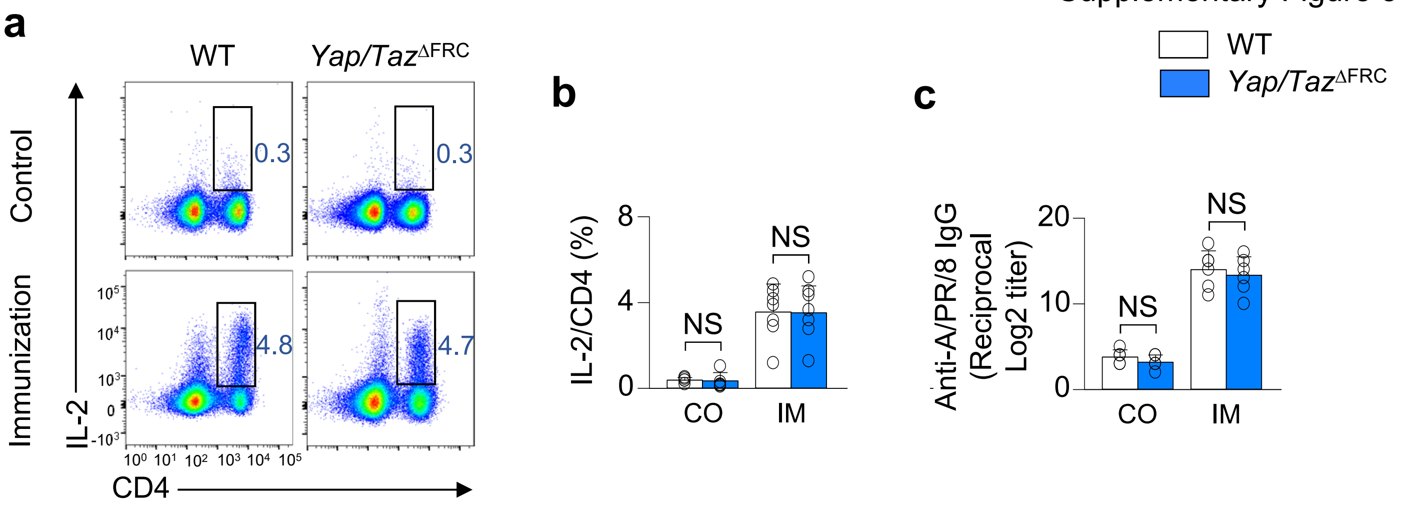

Supplementary Figure 5: Antiviral immune responses in Yap/Taz$^{\Delta FRC}$ mice.

a, **b**,Flow cytometric analyses and comparisons of IL-2+CD4^{+} T cells in gated CD3ε^{+} T cells. CO, Control; IM, Immunized group. Each dot indicates a value obtained from one mouse and *n* = 5 (CO) or 7 (IM) mice from two independent experiments.

c, Comparisons of anti-A/PR/8 IgG Ab titer in serum. Each dot indicates a value obtained from one mouse and *n* = 5 (CO) or *n* = 6 (IM) mice from two independent experiments. Horizontal bars indicate mean ± SD and *P* values versus WT by two-tailed Mann-Whitney *U* test. NS, not significant.

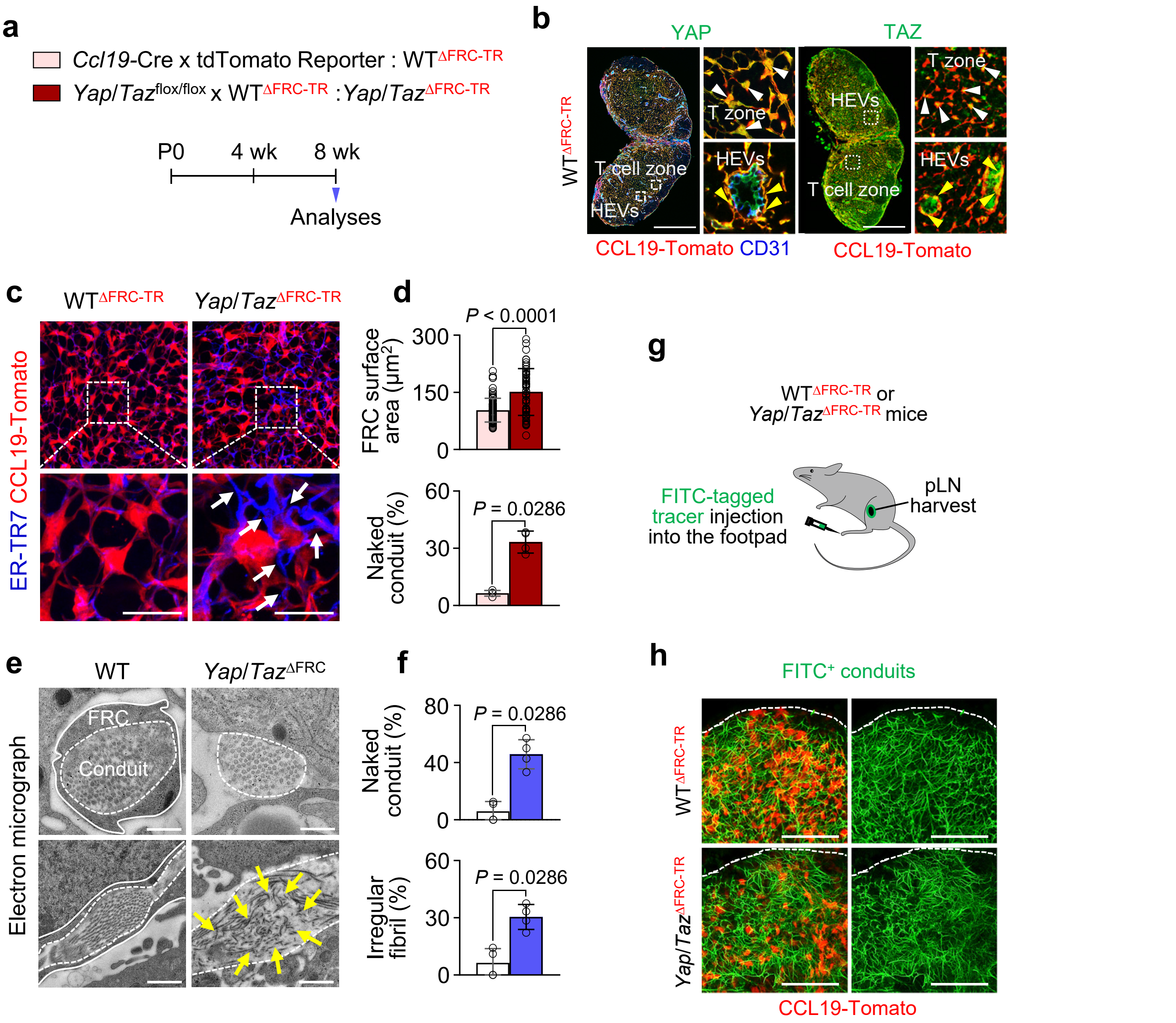

Supplementary Figure 6: Minor structural defects in LN conduit system in *Yap/Taz*$^{\Delta FRC}$ mice.

a, Diagram for generation of indicated mice and their analyses at 8 weeks after birth.

b, Representative images of YAP and TAZ expressions in CCL19^{+} FRCs of inguinal LN in 8-weeks-old adult WT$^{\Delta FRC\text{-}TR}$ mice. FRCs around T cell zone (white arrowheads) and high endothelial venules (HEVs, yellow arrowheads) are magnified as indicated. Scale bars, 500 μm. Similar findings were observed in *n* = 6 mice from three independent experiments.

c,**d**, Representative images and comparisons of FRC surface area and LN conduit coverage in WT and *Yap*/*Taz*$^{\Delta FRC}$ mice. FRCs around the T cell zone of the iLN within the white dashed box is magnified below to show ER-TR^{+} naked conduit (white arrowheads). Scale bars, 20 μm. Each dot indicates a value of a single FRC from 3~4 mid-sections/LN using *n* = 4 mice/group for surface area analysis or a mean value obtained from one mouse using *n* = 4 mice/group for conduit coverage analysis both from two independent experiments. Horizontal bars indicate mean ± SD and *P* values versus WT by two-tailed Mann-Whitney *U* test.

e,**f**, Representative electron microscope images and comparisons of conduit coverage by FRCs and regularity of collagen fibrils within the conduit in WT and *Yap*/*Taz*$^{\Delta FRC}$ mice. Scale bars, 500 nm. Each dot indicates a mean value obtained from one mouse and n = 4 mice/group pooled from two independent experiments. Horizontal bars indicate mean ± SD and *P* values versus WT by two-tailed Mann-Whitney *U* test.

g, Diagram depicting the experiment scheme for examining conduit functionality in WT$^{\Delta FRC\text{-}TR}$ and *Yap*/*Taz*$^{\Delta FRC\text{-}TR}$ mice. Draining popliteal LNs were analyzed 10 min after the subcutaneous injection of 10 μL of saturated FITC solution (0.1 mg/mL in HBSS) into the unilateral footpad.

h, Representative whole-mount images showing FITC^{+} conduits under the LN capsule (white dashed line) of popliteal LN in WT$^{\Delta FRC\text{-}TR}$ and *Yap*/*Taz*$^{\Delta FRC\text{-}TR}$ mice. Scale bars, 400 μm. Similar findings were observed in n = 6 mice/group from three independent experiments.

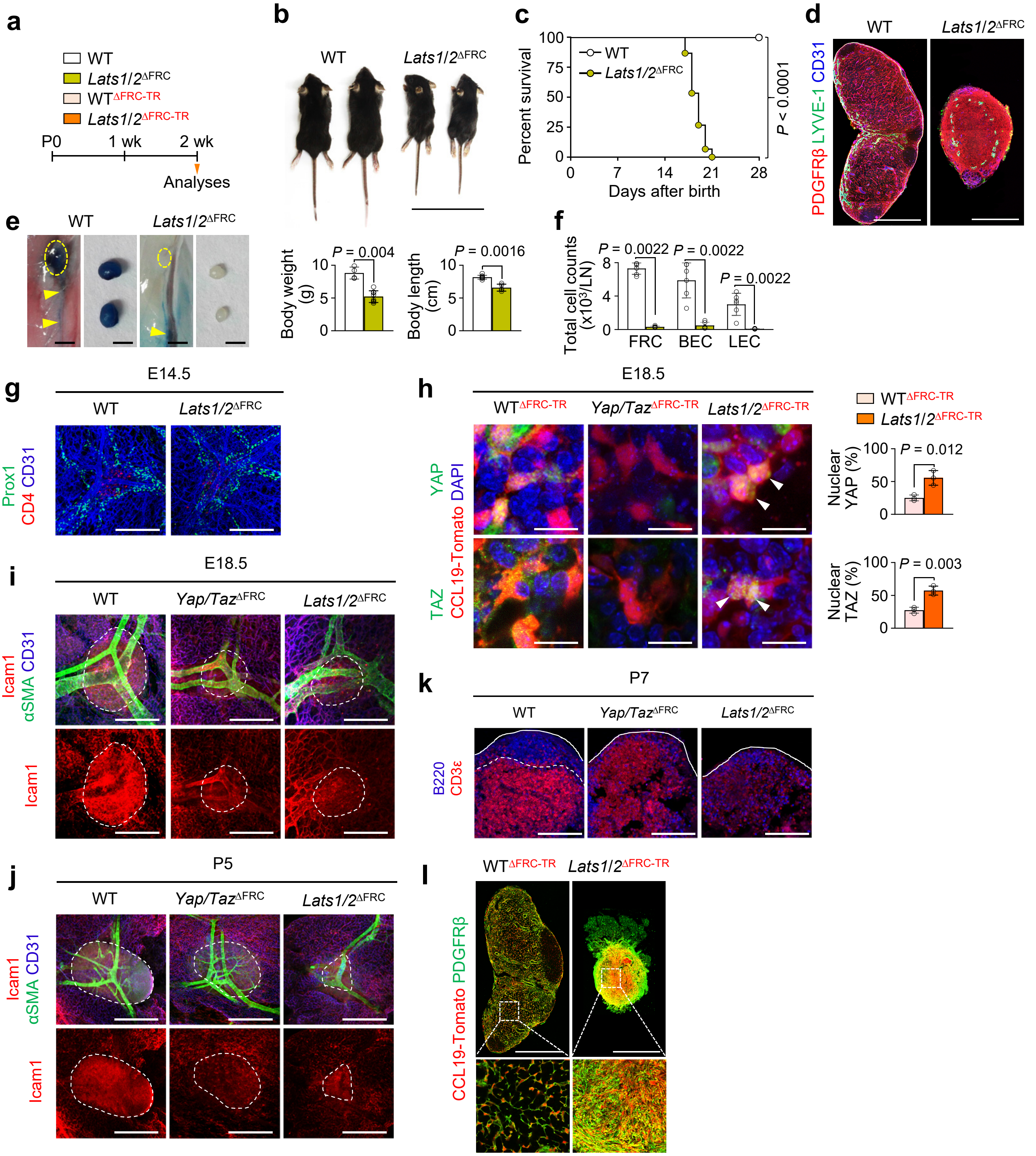
a
WT
Lats1/2ΔFRC
WTΔFRC-TR
Lats1/2ΔFRC-TR
P0
1 wk
2 wk
Analyses
b
WT
Lats1/2ΔFRC
P = 0.004
Body weight (g)
P = 0.0016
Body length (cm)
c
Percent survival
WT
Lats1/2ΔFRC
P < 0.0001
Days after birth
d
WT
Lats1/2ΔFRC
PDGFRβ LYVE-1 CD31
e
WT
Lats1/2ΔFRC
f
P = 0.0022
P = 0.0022
P = 0.0022
Total cell counts (x10^3/LN)
FRC
BEC
LEC
g
E14.5
WT
Lats1/2ΔFRC
Prox1 CD31
CD4
h
E18.5
WTΔFRC-TR
Yap/TazΔFRC-TR
Lats1/2ΔFRC-TR
YAP DAPI
CCL19-Tomato
TAZ
WTΔFRC-TR
Lats1/2ΔFRC-TR
Nuclear YAP (%)
P = 0.012
Nuclear TAZ (%)
P = 0.003
i
E18.5
WT
Yap/TazΔFRC
Lats1/2ΔFRC
Icam1
αSMA CD31
Icam1
k
P7
WT
Yap/TazΔFRC
Lats1/2ΔFRC
B220
CD3ε
j
P5
WT
Yap/TazΔFRC
Lats1/2ΔFRC
Icam1
αSMA CD31
Icam1
l
WTΔFRC-TR
Lats1/2ΔFRC-TR
CCL19-Tomato PDGFRβ

Supplementary Figure 7: Proper activation of YAP/TAZ is required for LN development.
a, Diagram for analyses of indicated mice at 2 weeks after birth.
b, Gross appearance and comparisons of body weight and body length in WT and *Lats1*/2$^{\Delta FRC}$ mice (showing from top to bottom). Scale bar, 5 cm. Each dot indicates a value obtained from one mouse and *n* = 4 (WT) or *n* = 8 (*Lats1*/2$^{\Delta FRC}$) mice from two independent experiments.
c, Kaplan-Meier survival analysis of WT and *Lats1*/2$^{\Delta FRC}$ mice. Each dot indicates a survival of one mouse and dots are overlapped from *n* = 15 mice/group pooled from two independent experiments. *P* value versus WT by log-rank test.
d, Representative images of inguinal LN showing LYVE-1$^+$ lymphatic vessels and CD31$^+$ blood vessels in WT and *Lats1*/2$^{\Delta FRC}$ mice. Scale bars, 500 µm. Similar findings were observed in *n* = 6 mice from two independent experiments.
e, Representative gross images showing drainage of Evans blue dye (yellow arrowheads) into the draining popliteal LN (white dotted lined circles) at 30 min after the dye injection into the bilateral footpad in WT and *Lats1/2* $^{\Delta FRC}$ mice. Isolated popliteal LNs are shown in the right panel. Scale bars, 1 mm. Similar findings were observed in *n* = 6 mice from two independent experiments.
f, Comparisons of total number of indicated stromal cells within the skin-draining LNs in WT and *Lats1/2* $^{\Delta FRC}$ mice. Dot indicates values obtained from *n* = 6 mice/group.
g, Representative whole-mount images of developing inguinal LNs at E14.5 showing engagement of Prox1$^+$ LECs with CD4$^+$ LTi cells within the LN anlagen in WT and *Lats1*/2$^{\Delta FRC}$ mice. Scale bars, 200 µm. Similar findings were observed in n = 6 mice from three independent experiments.
h, Representative images and comparison of YAP/TAZ expression and nuclear localization (white arrowheads) in *Ccl19*-expressing LTo cells of inguinal LN at E18.5 in WT$^{\Delta FRC\text{-}TR}$, *Yap*/*Taz*$^{\Delta FRC\text{-}TR}$ and *Lats1*/2$^{\Delta FRC\text{-}TR}$ mice. Scale bars, 10 µm.
i, Representative whole-mount images of developing inguinal LNs at E18.5 showing Icam1$^+$ LTo cells, αSMA$^+$ smooth muscle cells, and CD31$^+$ blood vessels within the LN anlagen (demarcated with the white dashed-line) in WT$^{\Delta FRC\text{-}TR}$, *Yap*/*Taz*$^{\Delta FRC\text{-}TR}$, and *Lats1*/2$^{\Delta FRC\text{-}TR}$ mice. Scale bars, 500 µm.
j, Representative whole-mount images of inguinal LNs at P5 showing Icam1$^+$ LTo cells, αSMA$^+$ smooth muscle cells, and CD31$^+$ blood vessels within the LN anlagen (demarcated with the white dashed-line) in WT$^{\Delta FRC\text{-}TR}$, *Yap*/*Taz*$^{\Delta FRC\text{-}TR}$, and *Lats1*/2$^{\Delta FRC\text{-}TR}$ mice. Scale bars, 500 µm.
k, Representative images of border between B and T cell zones (white dashed line) beneath the inguinal LN capsule (white line) at P7 in WT$^{\Delta FRC\text{-}TR}$, *Yap*/*Taz*$^{\Delta FRC\text{-}TR}$, and *Lats1*/2$^{\Delta FRC\text{-}TR}$ mice. Scale bars, 100 µm.
l, Representative images of inguinal LN showing Tomato$^+$PDGFRβ$^+$ FRCs in WT$^{\Delta FRC\text{-}TR}$ and *Lats1*/2$^{\Delta FRC\text{-}TR}$ mice. The regions within the white dashed box are magnified as indicated. Scale bars, 500 µm. Similar findings were observed in *n* = 4 mice from two independent experiments.
Unless otherwise denoted, horizontal bars indicate mean ± SD and *P* value versus WT by two-tailed Mann-Whitney *U* test. NS, not significant.

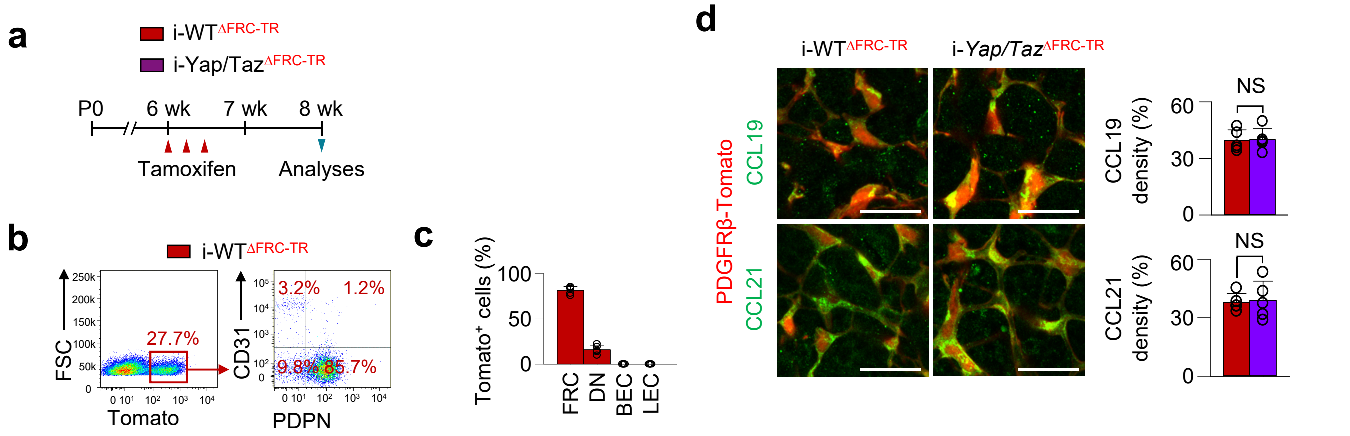

Supplementary Figure 8: YAP/TAZ is dispensable for chemokine expression in adult mature FRCs.
a, Diagram for generation of indicated mice for their analyses at 8-weeks-old after tamoxifen delivery from 6-weeks-old.
b,**c**, Representative flow cytometric plots gated from CD45$^-$ stromal cells of skin-draining LNs in i-*Yap*/*Taz*$^{\Delta FRC\text{-}TR}$ mice showing subpopulations of Tomato$^+$ cells. Each dot indicates a mean value obtained from one mouse and *n* = 5 mice/group pooled from two independent experiments.
d, Representative images and comparisons of indicated marker expressions in FRCs around T cell zone of inguinal LN in i-WT$^{\Delta FRC\text{-}TR}$ and i-*Yap*/*Taz*$^{\Delta FRC\text{-}TR}$ mice. Scale bars, 20 μm. Each dot indicates a value obtained from one mouse using n = 5 mice/group, pooled from two independent experiments. Horizontal bars indicate mean ± SD and P values versus i-WT$^{\Delta FRC\text{-}TR}$ by two-tailed Mann-Whitney U test. NS, not significant.

Supplementary Figure 9

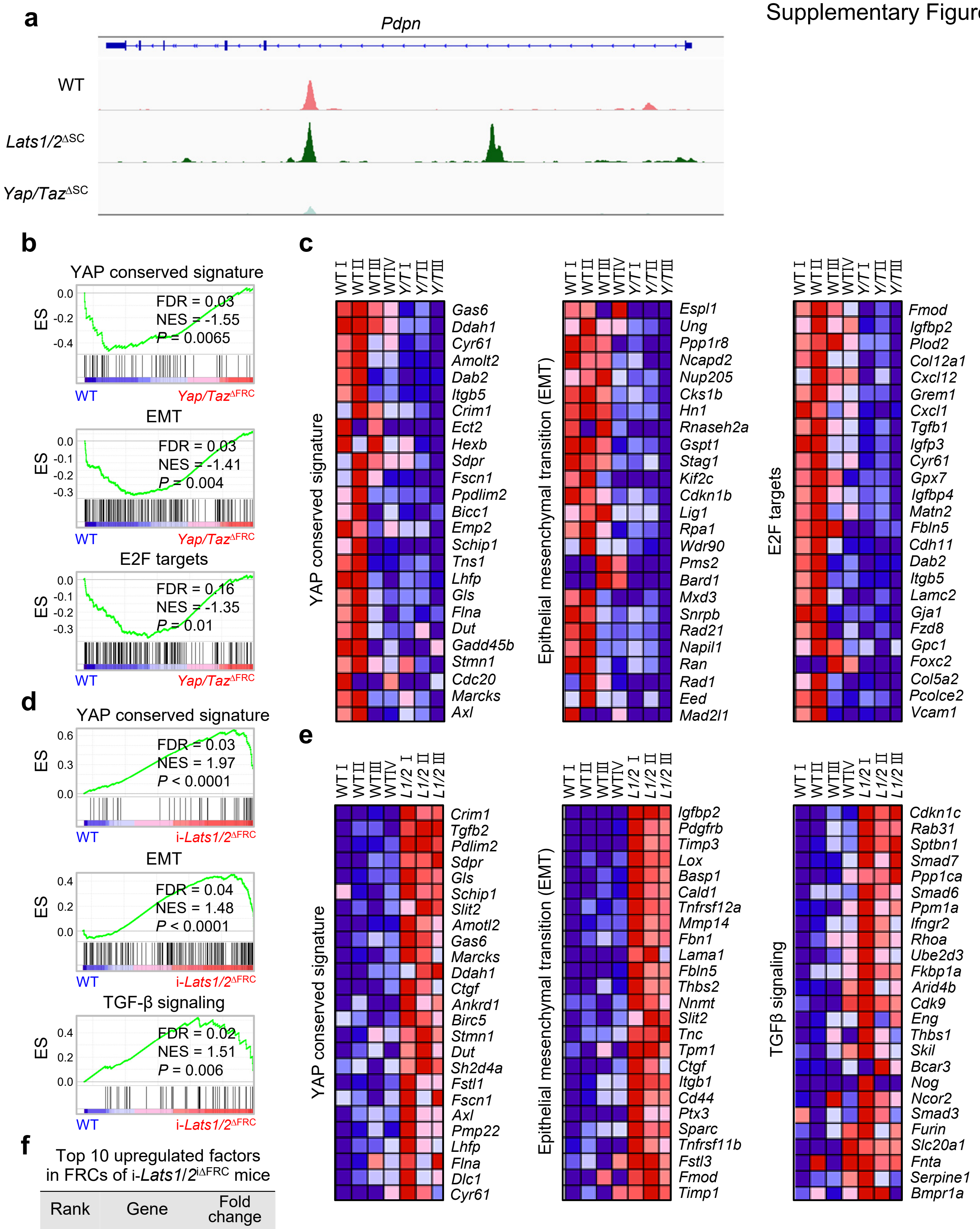

f

Top 10 upregulated factors in FRCs of i-$Lats1/2^{i\Delta FRC}$ mice

Rank	Gene	Fold change
1	*Wisp2*	78.0
2	*Nov*	57.3
3	*Fgf18*	32.3
4	*Bmp4*	15.7
5	*Bmp3*	7.4
6	*Tgfb2*	5.9
7	*Vegfd*	4.9
8	*Ogn*	4.2
9	*Ctgf*	3.1
10	*Pdgfa*	2.7

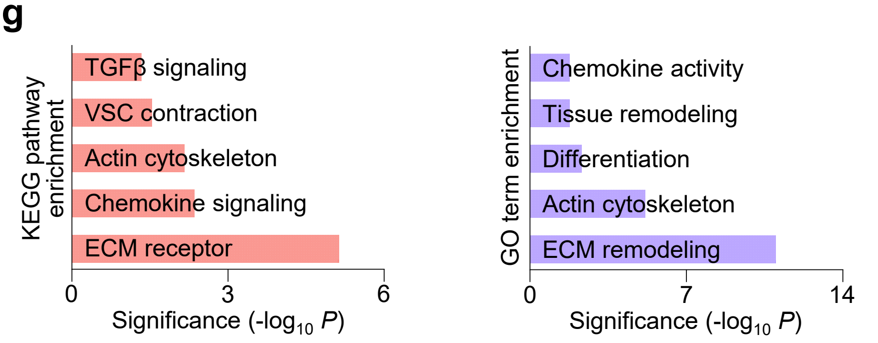

Supplementary Figure 9: Distinct transcriptomic profiles of FRCs by *Yap*/*Taz* depletion or hyperactivation.

a, Chromatin accessibility profiles of Lgr5^{+} stem cells (SC) showing representative genomic promoter region of *Pdpn* in WT (*Lgr5*$^{+}$ cre; tdtomato), *Lats1*/*2*$^{\Delta SC}$ (*Lgr5*$^{+}$ cre; tdtomato; *Lats1/2* fl/fl), and *Yap*/*Taz*$^{\Delta SC}$ (*Lgr5*$^{+}$ cre; tdtomato; *Yap/Taz* fl/fl) mice.

b,**c**, GSEA of FRCs in *Yap*/*Taz*$^{\Delta FRC}$ mice compared with WT and corresponding heatmaps of top 25 enriched genes.

d,**e**, GSEA of FRCs in i-*Lats1*/*2*$^{\Delta FRC}$ mice compared with WT and corresponding heatmaps of top 25 enriched genes.

f, Ranking list of top 10 upregulated growth factors in FRCs of i-*Lats1*/*2*$^{\Delta FRC}$ mice compared with WT.

g, KEGG pathway and GO term enrichment analysis of 1,152 differentially expressed genes of FRC in i-*Lats1*/*2*$^{\Delta FRC}$ mice compared with WT. Ranked according to the significance of *P* value ($-\log_{10} P$). VSC, vascular smooth muscle cells; ECM, extracellular matrix.

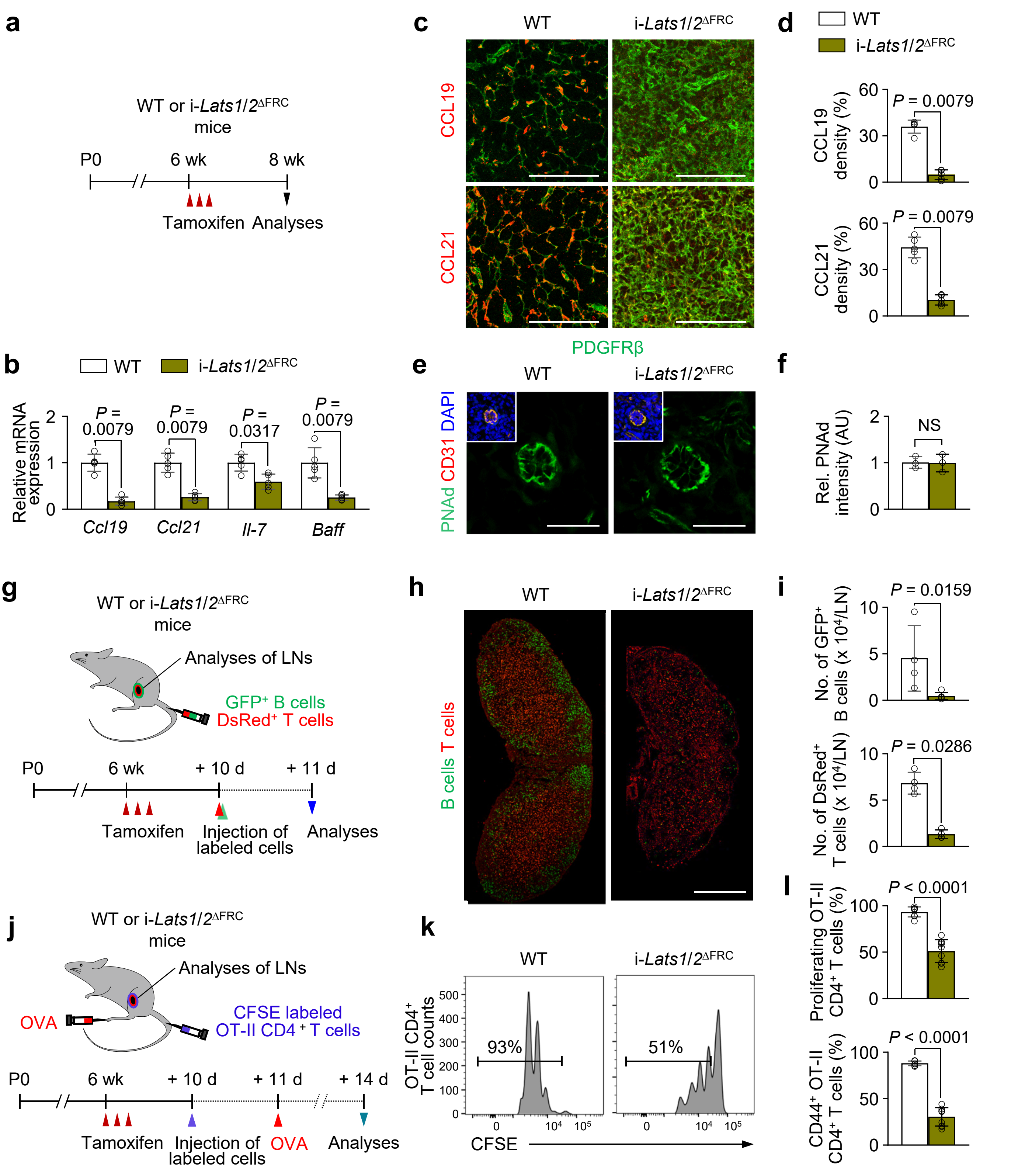
a
WT or i-Lats1/2ΔFRC mice
P0
6 wk
8 wk
Tamoxifen
Analyses
b
WT
i-Lats1/2ΔFRC
Relative mRNA expression
P = 0.0079
P = 0.0079
P = 0.0317
P = 0.0079
Ccl19
Ccl21
Il-7
Baff
c
WT
i-Lats1/2ΔFRC
CCL19
CCL21
PDGFRβ
d
CCL19 density (%)
CCL21 density (%)
P = 0.0079
P = 0.0079
e
PNAd CD31 DAPI
f
Rel. PNAd intensity (AU)
NS
g
WT or i-Lats1/2ΔFRC mice
Analyses of LNs
GFP+ B cells
DsRed+ T cells
P0
6 wk
+ 10 d
+ 11 d
Tamoxifen
Injection of labeled cells
Analyses
h
B cells T cells
i
No. of GFP+ B cells (x 10^4/LN)
P = 0.0159
No. of DsRed+ T cells (x 10^4/LN)
P = 0.0286
j
WT or i-Lats1/2ΔFRC mice
Analyses of LNs
OVA
CFSE labeled OT-II CD4+ T cells
P0
6 wk
+ 10 d
+ 11 d
+ 14 d
Tamoxifen
Injection of labeled cells
OVA
Analyses
k
WT
i-Lats1/2ΔFRC
OT-II CD4+ T cell counts
93%
51%
CFSE
l
Proliferating OT-II CD4+ T cells (%)
P < 0.0001
CD44+ OT-II CD4+ T cells (%)
P < 0.0001

Supplementary Figure 10: YAP/TAZ hyperactivation in FRCs impairs adaptive immune response.
a, Diagram for analyses of indicated mice at 8-weeks-old after the tamoxifen delivery from 6-weeks-old.
b, Comparison of indicated mRNA expression in FRCs sorted from WT and i-*Lats1/2*$^{\Delta FRC}$ mice. Each dot indicates a mean of pentuiplicate values using *n* = 10~15 mice/group from three independent experiments.
c,**d**, Representative images and comparisons of densities of CCL19 and CCL21 in PDGFRβ^{+} FRCs at T cell zone of inguinal LN in WT and i-*Lats1/2*$^{\Delta FRC}$ mice. Scale bars, 100 μm. Each dot indicates a mean value obtained from one mouse and *n* = 5 mice/group pooled from two independent experiments.
e,**f**, Representative images and comparison of PNAd expression on HEVs of inguinal LN in WT and i-*Lats1/2*$^{\Delta FRC}$ mice. Single channel PNAd^{+} image is magnified from the inset at the top left corner. Scale bars, 10 μm. Each dot indicates a mean value obtained from one mouse and *n* = 3 mice/group pooled from two independent experiments.
g, Diagram depicting the experimental scheme for adaptive transfer of GFP^{+} B cells and DsRed^{+} T cells in tamoxifen delivered WT and i-*Lats1/2*$^{\Delta FRC}$ mice for their analyses at the indicated timepoint counting from the day of tamoxifen injection.
h,**i**, Representative images and comparisons of homing of transferred GFP^{+} B cells and DsRed^{+} T cells in inguinal LNs of WT and i-*Lats1/2*$^{\Delta FRC}$ mice. Scale bars, 500 μm. Each dot indicates a value obtained from one mouse using *n* = 4 (WT) or *n* = 5 (i-*Lats1/2*$^{\Delta FRC}$) mice for comparison of GFP^{+} B cells and *n* = 4 mice/group for comparison of DsRed^{+} T cells, both pooled from two independent experiments.
j, Diagram depicting the experimental scheme for adaptive transfer of CFSE-labeled OT-II CD4^{+} T cells and OVA injection into the footpad in WT and i-*Lats1/2*$^{\Delta FRC}$ mice for their analyses at the indicated time point counting from the day of tamoxifen injection.
k,**l**, Representative plots and comparisons of proliferation and CD44 expression of the transferred OT-II CD4^{+} T cells in WT and i-*Lats1/2*$^{\Delta FRC}$ mice. Percentages of proliferating cells analyzed by CFSE dilution are indicated above bracketed lines. Each dot indicates a mean value obtained from one mouse using *n* = 8 (WT) or *n* = 9 (i-*Lats1/2*$^{\Delta FRC}$) mice pooled from two independent experiments.
Unless otherwise denoted, horizontal bars indicate mean ± SD and *P* value versus WT by two-tailed Mann-Whitney *U* test. NS, not significant.

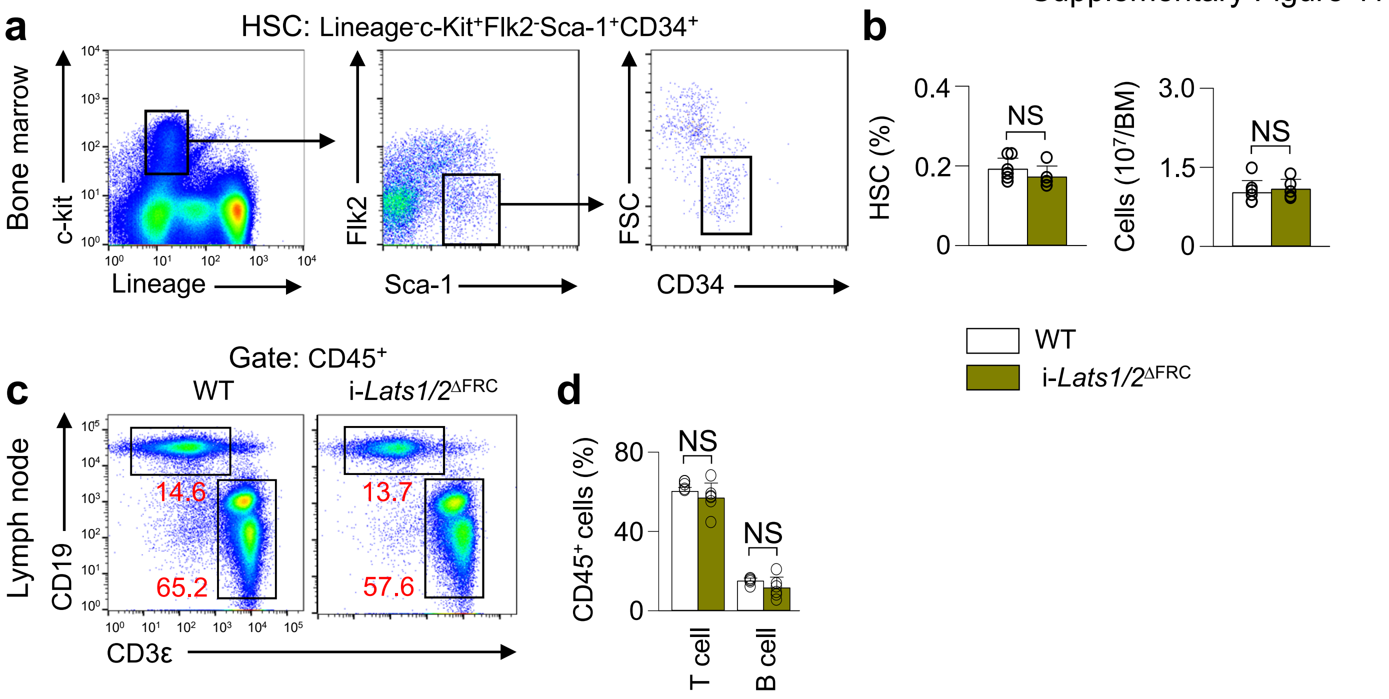

Supplementary Figure 11: No apparent differences in immune cell composition in bone marrow and lymph node of i-*Lats1/2*$^{\Delta FRC}$ mice.

BM, LNs and peripheral blood from 8-week-old WT and i-*Lats1/2*$^{\Delta FRC}$ mice were sampled and analyzed.

a, **b**, Flow cytometric plots gated for c-Kit^{+}Flk2^{-}Sca1^{+}CD34^{+} HSC and comparisons of percentage of HSC and cellularity of BM. Each dot indicates a value obtained from one mouse and *n* = 7 (WT) or 5 (i-*Lats1/2*$^{\Delta FRC}$) mice from two independent experiments. Horizontal bars indicate mean ± SD and *P* values versus WT by two-tailed Mann-Whitney *U* test. NS, not significant.

c, **d**, Flow cytometric plots and comparisons of T and B lymphocytes which are pre-gated as CD45^{+} cells in LNs. Numbers indicate percentages of CD19^{-}CD3ε^{+} T cells and CD19^{+}CD3ε^{-} B cells. Dot indicates values obtained from *n* = 6 mice/group. Horizontal bars indicate mean ± SD and *P* values versus WT by two-tailed Mann-Whitney *U* test.NS, not significant.

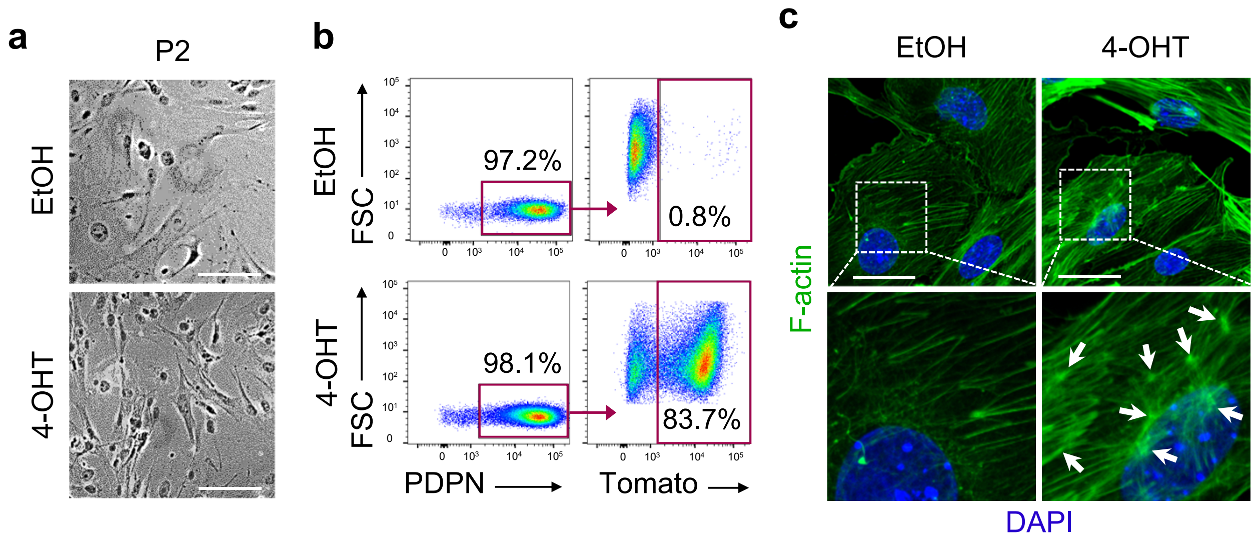

Supplementary Figure 12: YAP/TAZ hyperactivation potentiates contraction of cultured mouse FRCs.
a, Representative phase contrast images of primary cultured mouse FRCs at passage (P) 2 after treatment with EtOH or 4-OHT for 2 d. Scale bars, 100 μm. Similar findings were observed in three independent experiments.
b, Representative flow cytometric plots of Tomato⁺ FRCs gated from PDPN⁺ cells of primary cultured mouse FRCs after treatment with EtOH or 4-OHT. Similar findings were observed in three independent experiments.
c, Representative images of F-actin filaments in primary cultured mouse after treatment with EtOH or 4-OHT for 2 d. White dashed box is magnified in the lower panel to show enhanced assembly of F-actin filaments (white arrowheads). Scale bars, 30 μm. Similar findings were observed in three independent experiments.

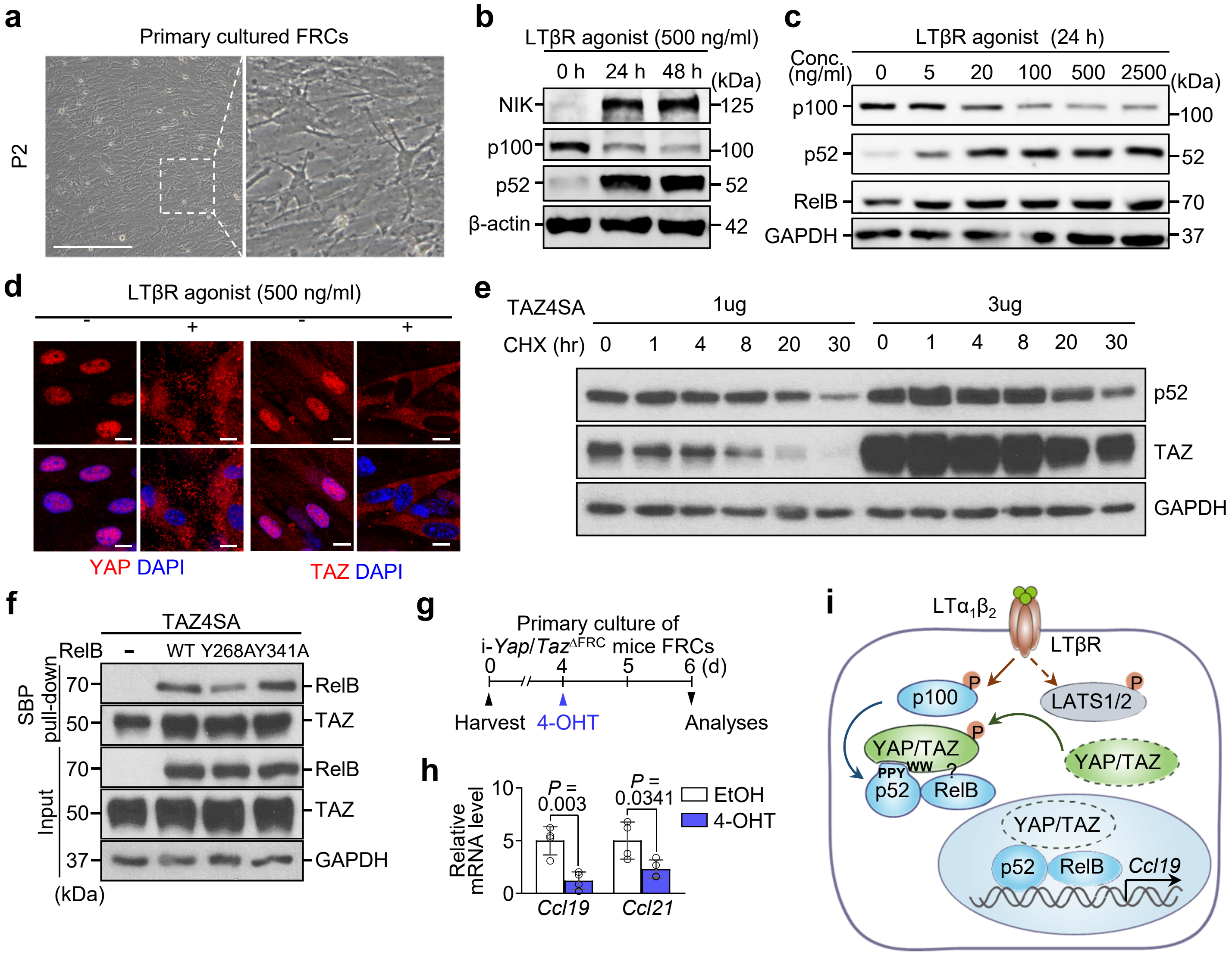

Supplementary Figure 13: LTβR activation promotes cytoplasmic translocation of YAP/TAZ-p52.
a, Phase contrast image of primary cultured FRCs. The region within the white dashed box is magnified in the right panel. Scale bar, 200 µm.
b, Immunoblot analysis of indicated proteins in primary cultured FRCs after stimulation with LTβR agonistic antibody (500 ng/ml) for indicated time points. Similar findings were observed in three independent experiments.
c Immunoblot analysis of indicated proteins in MEFs after stimulation with LTβR agonistic antibody at the indicated concentration for 24 h. Similar findings were observed in three independent experiments.
d, Representative images of YAP or TAZ after treatment with or without LTβR agonistic antibody (500 ng/ml) for 5 d in cultured FRCs derived from WT mice. Nuclei are stained with DAPI. Scale bars, 50 µm. Similar findings were observed in three independent experiments.
e, Immunoblot analysis of indicated proteins in HEK-293T cells after transfection with the streptavidin-binding peptide (SBP)-TAZ4SA with p52, and protein synthesis was blocked by treatment of 50µg/ml cycloheximide (CHX) for indicated time point. p52 level were monitored by western blot. Similar findings were observed in three independent experiments.
f, Pull-down assay with streptavidin resin in HEK-293T cells after transfection with the streptavidin-binding peptide (SBP)-TAZ4SA with plasmids encoding empty vector (-), RelB (WT), and RelB mutants (Y268A and Y341A) and immunoblot analysis with anti-RelB, anti-TAZ, anti-GAPDH antibodies. Similar findings were observed in three independent experiments.
g, Diagram for primary culture of FRCs derived from i-*Yap*/*Taz*$^{\Delta FRC}$ mice and treatment with EtOH (control) or 4-OHT for their analyses at the indicated time point.
h, Comparison of indicated mRNA expression in primary cultured FRCs after treatment with EtOH or 4-OHT. Each dot indicates a mean of quadruplicate values from two independent experiments. Horizontal bars indicate mean ± SD and *P* values versus EtOH by two-tailed Student's t-test.
i, Schematic image depicting the role of LTβR in promoting Hippo signaling by p52-YAP/TAZ interaction and their cytoplasmic retention. Bronze circled 'P's indicate phosphorylation sites of p100, LATS1/2 (T1079), and YAP (S127).

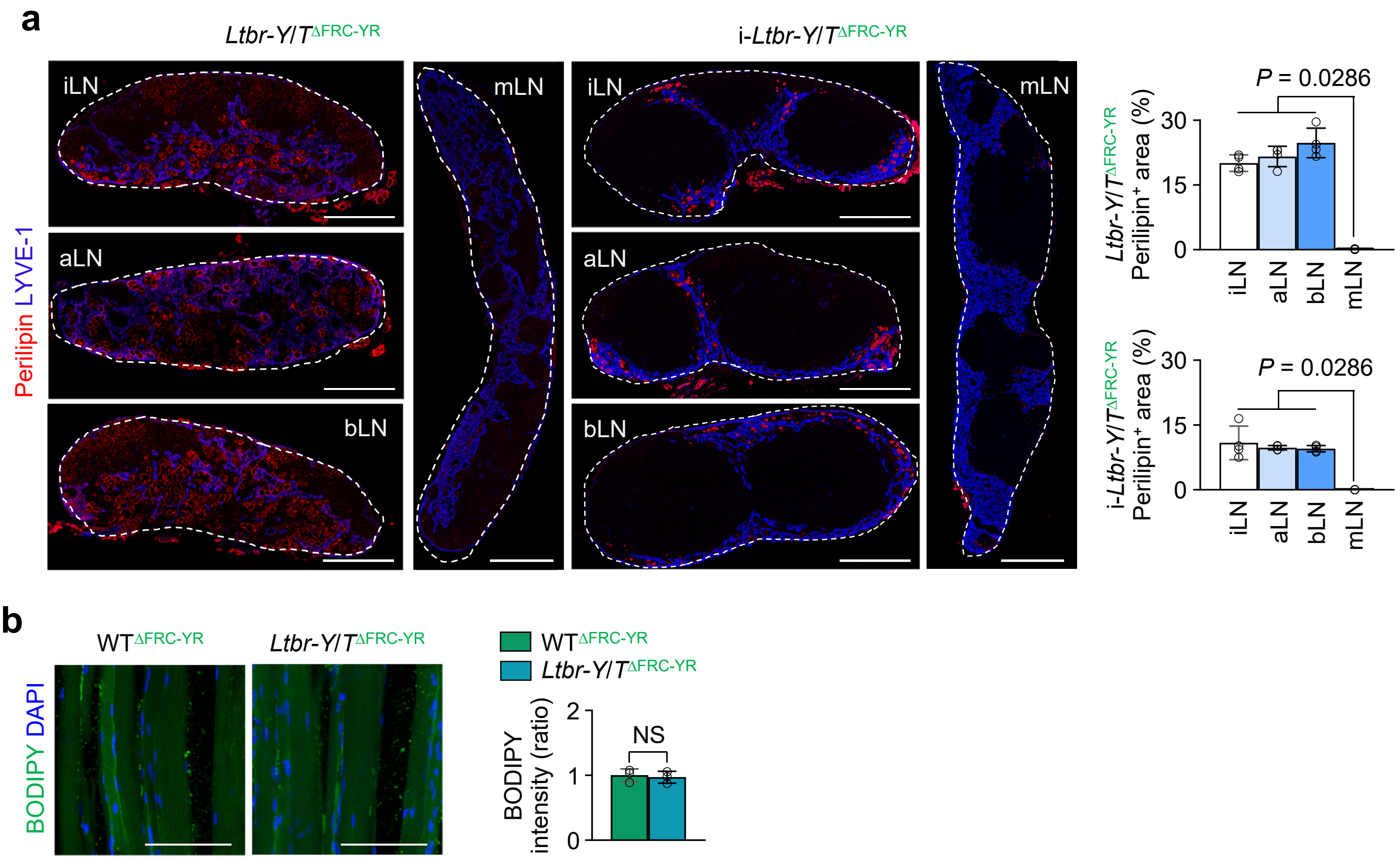

Supplementary Figure 14: FRCs transform into adipocytes preferentially in skin-draining LNs by triple depletion of *Ltbr* and *Yap*/*Taz*.

a, Representative images and comparison of perilipin$^+$ adipocyte area in inguinal LN (iLN), axillary LN (aLN), brachial LN (bLN), and mesenteric LN (mLN) of *Ltbr-Y*/*T*$^{ΔFRC-YR}$ or i-*Ltbr-Y*/*T*$^{ΔFRC-YR}$ mice. Scale bars, 200 μm. Each dot indicates a mean value obtained from one mouse and n = 4 mice/group pooled from two independent experiments. Horizontal bars indicate mean ± SD and P values versus iLN, aLN, or bLN by two-tailed Mann-Whitney U test.

b, Representative images and comparison of BODIPY$^+$ lipid droplets within the skeletal muscle (quadriceps) in WT$^{ΔFRC-YR}$ and *Ltbr-Y*/*T*$^{ΔFRC-YR}$ mice. Scale bars, 100 μm. Each dot indicates a mean value obtained from one mouse and n = 3 mice/group pooled from two independent experiments. Horizontal bars indicate mean ± SD. NS, not significant.

Supplementary Table 1. List of mouse model nomenclatures

Symbol	Nomenclature	Allele	
	WT	Cre	-
		Flox	*Yap/Taz*$^{flox/flox}$; *Lats1/2*$^{flox/flox}$; *Lats1/2*$^{flox/flox}$ x *Yap/Taz*$^{flox/flox}$; *Ltbr*$^{flox/flox}$
	Yap/Taz$^{\Delta FRC}$	Cre	*Ccl19*-Cre
		Flox	*Yap/Taz*$^{flox/flox}$
	Yap$^{\Delta FRC}$	Cre	*Ccl19*-Cre
		Flox	*Yap*$^{flox/flox}$
	Taz$^{\Delta FRC}$	Cre	*Ccl19*-Cre
		Flox	*Taz*$^{flox/flox}$
	WT$^{\Delta FRC\text{-}TR}$	Cre	*Ccl19*-Cre
		Flox	tdTomato Reporter
	Yap/Taz$^{\Delta FRC\text{-}TR}$	Cre	*Ccl19*-Cre
		Flox	*Yap/Taz*$^{flox/flox}$ x tdTomato
	Lats1/2$^{\Delta FRC}$	Cre	*Ccl19*-Cre
		Flox	*Lats1/2*$^{flox/flox}$
	Lats1/2$^{\Delta FRC\text{-}TR}$	Cre	*Ccl19*-Cre
		Flox	*Lats1/2*$^{flox/flox}$ x tdTomato
-	*L1/2-Y/T*$^{\Delta FRC}$	Cre	*Ccl19*-Cre
		Flox	*Lats1/2*$^{flox/flox}$ x *Yap/Taz*$^{flox/flox}$
	i-WT$^{\Delta FRC\text{-}TR}$	Cre	*Pdgfrb*-CreER^{T2}
		Flox	tdTomato
	i-*Yap/Taz*$^{\Delta FRC\text{-}TR}$	Cre	*Pdgfrb*-CreER^{T2}
		Flox	*Yap/Taz*$^{flox/flox}$ x tdTomato
	i-*Lats1/2*$^{\Delta FRC}$	Cre	*Pdgfrb*-CreER^{T2}
		Flox	*Lats1/2*$^{flox/flox}$
	i-*L1/2-Y/T*$^{\Delta FRC}$	Cre	*Pdgfrb*-CreER^{T2}
		Flox	*Lats1/2*$^{i\Delta FRC}$ x *Yap/Taz*$^{flox/flox}$
	Ltbr$^{\Delta FRC}$	Cre	*Ccl19*-Cre
		Flox	*Ltbr*$^{flox/flox}$
	WT$^{\Delta FRC\text{-}YR}$	Cre	*Ccl19*-Cre
		Flox	YFP
	Ltbr$^{\Delta FRC\text{-}YR}$	Cre	*Ccl19*-Cre
		Flox	*Ltbr*$^{flox/flox}$ x YFP
-	*Y/T*$^{\Delta FRC\text{-}YR}$	Cre	*Ccl19*-Cre
		Flox	*Yap/Taz*$^{flox/flox}$ x YFP
	Ltbr-Y/T$^{\Delta FRC\text{-}YR}$	Cre	*Ccl19*-Cre
		Flox	*Ltbr*$^{flox/flox}$ x *Yap/Taz*$^{flox/flox}$ x YFP
-	i-WT$^{\Delta FRC\text{-}YR}$	Cre	*Pdgfrb*-CreER^{T2}
		Flox	*Ltbr*$^{flox/flox}$ x YFP
-	i-*Y/T*$^{\Delta FRC\text{-}YR}$	Cre	*Pdgfrb*-CreER^{T2}
		Flox	*Yap/Taz*$^{flox/flox}$ x YFP
-	i-*Ltbr*$^{\Delta FRC\text{-}YR}$	Cre	*Pdgfrb*-CreER^{T2}
		Flox	*Ltbr*$^{flox/flox}$ x YFP
-	i-*Ltbr-Y/T*$^{\Delta FRC\text{-}YR}$	Cre	*Pdgfrb*-CreER^{T2}
		Flox	*Ltbr*$^{flox/flox}$ x *Yap/Taz*$^{flox/flox}$ x YFP

Supplementary Table 2. Mouse primer sets for quantitative RT-PCR, luciferase reporter, and ChIP construct.

Name		Sequence (5'-3')
Gapdh	Forward	TGTTCCTACCCCCAATGTGT
	Reverse	TGTGAGGGAGATGCTCAGTG
Acta2	Forward	AGGGCTGTTTTCCCATCCATCG
	Reverse	TCTCTTGCTCTGGGCTTCATCC
Amotl2	Forward	AGAGATTGGAATCGGCAAAC
	Reverse	TTCTCCTGTTCCTGTTGCTG
Ankrd1	Forward	GCTGGTAACAGGCAAAAAGAAC
	Reverse	CCTCTCGCAGTTTCTCGCT
Baff	Forward	TGCCTTGGAGGAGAAAGAGA
	Reverse	GGAATTGTTGGGCAGTGTTT
Ccl19	Forward	CTGCCTCAGATTATCTGCCAT
	Reverse	GTCTTCCGCATCATTAGCAC
Ccl21	Forward	ATCCCGGCAATCCTGTTCTC
	Reverse	GGTTCTGCACCCAGCCTTC
Ctgf	Forward	GTGCCAGAACGCACACTG
	Reverse	CCCCGGTTACACTCCAAA
Cyr61	Forward	ATGATGATCCAGTCCTGCAA
	Reverse	TAGGCTGTACAGTCGGAACG
Il7	Forward	GTGCCACATTAAAGACAAAGAAG
	Reverse	GTTCATTATTCGGGCAATTACTATC
Pparg	Forward	TCGCTGATGCACTGCCTATG
	Reverse	GAGAGGTCCACAGAGCTGATT
Cebpa	Forward	GCGGGAACGCAACAACATC
	Reverse	GTCACTGGTCAACTCCAGCAC
Luc_mCcl19_NF2	Forward	TTTGCTAGC TCACGCTTGCTCTGAAGGTA
	Reverse	TTTTCTCGAGCACAGGGGAGTGACTGGAAT
Ccl19 -60b_NFKB2	Forward	GGGGAAGACCCAGAACGGGCCATCGAG
	Reverse	GCCTGCTGAGGCTGCCACCAGCTCC
