# Supplementary material for: YAP/TAZ direct commitment and maturation of lymph node fibroblastic reticular cells
Source: Nat Commun. 2020 Jan 24;11:519. doi: 10.1038/s41467-020-14293-1 (PMC6981200; doi:10.1038/s41467-020-14293-1)
Supplement: Supplementary file 2 — Reporting Summary [file 41467_2020_14293_MOESM2_ESM.pdf]

nature research

Corresponding author(s): Dae-Sik Lim and Gou Young Koh

Last updated by author(s): Dec 18, 2019

Reporting Summary

Nature Research wishes to improve the reproducibility of the work that we publish. This form provides structure for consistency and transparency in reporting. For further information on Nature Research policies, seeAuthors & Referees and theEditorial Policy Checklist .

Statistics

For all statistical analyses, confirm that the following items are present in the figure legend, table legend, main text, or Methods section.

n/a	Confirmed	
☐	☒	The exact sample size (*n*) for each experimental group/condition, given as a discrete number and unit of measurement
☐	☒	A statement on whether measurements were taken from distinct samples or whether the same sample was measured repeatedly
☐	☒	The statistical test(s) used AND whether they are one- or two-sided<br>*Only common tests should be described solely by name; describe more complex techniques in the Methods section.*
☐	☒	A description of all covariates tested
☐	☒	A description of any assumptions or corrections, such as tests of normality and adjustment for multiple comparisons
☐	☒	A full description of the statistical parameters including central tendency (e.g. means) or other basic estimates (e.g. regression coefficient) AND variation (e.g. standard deviation) or associated estimates of uncertainty (e.g. confidence intervals)
☐	☒	For null hypothesis testing, the test statistic (e.g. *F*, *t*, *r*) with confidence intervals, effect sizes, degrees of freedom and *P* value noted<br>*Give P values as exact values whenever suitable.*
☒	☐	For Bayesian analysis, information on the choice of priors and Markov chain Monte Carlo settings
☒	☐	For hierarchical and complex designs, identification of the appropriate level for tests and full reporting of outcomes
☒	☐	Estimates of effect sizes (e.g. Cohen's *d*, Pearson's *r*), indicating how they were calculated

Our web collection on statistics for biologists contains articles on many of the points above.

Software and code

Policy information about availability of computer code

Data collection	The following software were used for data collection:<br>LSM image software (Carl Zeiss)<br>Zen 2.3 software (Carl Zeiss)<br>FACS DIVA v8.0.1 (BD)
Data analysis	The following software were used for data analysis:<br>Zen 2.3 software (Carl Zeiss)<br>ImageJ Software (NIH)<br>FlowJo (Treestar)<br>GraphPad Prism 8.0 (GraphPad Software)<br>Imaris (Bitplane)<br>Ingenuity Pathway Analysis (Qiagen)<br>Gene Set Enrichment Analysis (Broad Institute)<br>Multiple Experiment Viewer (TIGF)<br>Cluster 3.0 (Eisen Labotatory)<br>TreeView (Eisen Labotatory)

For manuscripts utilizing custom algorithms or software that are central to the research but not yet described in published literature, software must be made available to editors/reviewers. We strongly encourage code deposition in a community repository (e.g. GitHub). See the Nature Research guidelines for submitting code & software for further information.

Data

Policy information about availability of data

All manuscripts must include a data availability statement. This statement should provide the following information, where applicable:

- Accession codes, unique identifiers, or web links for publicly available datasets
- A list of figures that have associated raw data
- A description of any restrictions on data availability

RNA sequencing data that support the findings of this study are deposited in the Gene Expression Omnibus (GSE89742).
Source data are provided as a Source Data file.
All other data supporting the findings of this study are available from the corresponding author on reasonable request.

Field-specific reporting

Please select the one below that is the best fit for your research. If you are not sure, read the appropriate sections before making your selection.

[x] Life sciences   [ ] Behavioural & social sciences   [ ] Ecological, evolutionary & environmental sciences

For a reference copy of the document with all sections, see nature.com/documents/nr-reporting-summary-flat.pdf

Life sciences study design

All studies must disclose on these points even when the disclosure is negative.

Sample size	Sample size was chosen in accordance with similar previously published experiments.
Data exclusions	No samples were excluded from the analysis.
Replication	More than three independent experiments were replicated for all analyses.
Randomization	Animals from different cages, but within the same experimental group, were selected to assure randomization.
Blinding	The investigators were blinded during the experiments and quantifications.

Reporting for specific materials, systems and methods

We require information from authors about some types of materials, experimental systems and methods used in many studies. Here, indicate whether each material, system or method listed is relevant to your study. If you are not sure if a list item applies to your research, read the appropriate section before selecting a response.

Materials & experimental systems

n/a	Involved in the study	
[ ]	[x]	Antibodies
[ ]	[x]	Eukaryotic cell lines
[x]	[ ]	Palaeontology
[ ]	[x]	Animals and other organisms
[ ]	[x]	Human research participants
[x]	[ ]	Clinical data

Methods

n/a	Involved in the study	
[x]	[ ]	ChIP-seq
[ ]	[x]	Flow cytometry
[x]	[ ]	MRI-based neuroimaging

Antibodies

Antibodies used	The following primary and secondary antibodies were used in the immunostaining: anti-YAP (rabbit monoclonal, D8H1X, Cell Signaling), anti-TAZ (rabbit polyclonal, HPA007415, Sigma-Aldrich), anti-PDPN (syrian hamster monoclonal, 127402, Biolegend), anti-CCL19 (goat polyclonal, PA5-47958, Thermo Fisher), anti-CCL21 (goat polyclonal, AF457, R&D), anti-ER-TR7 (rat monoclonal, sc-73355, Santa Cruz), anti-CD3e (hamster monoclonal, 145-2C11, BD), anti-B220 (rat monoclonal, RA3-6B2, BD), anti-CD31 (hamster monoclonal, 2H8, Millipore), anti-PDGFRβ (rat monoclonal, APB5, eBioscience), anti-Collagen IV (rabbit polyclonal, ab6586, Abcam), anti-Ki-67 (rabbit monoclonal, SP6, Abcam), anti-LYVE-1 (rabbit polyclonal, 11-034, Angiobio), anti-Caspase-3 (rabbit polyclonal, 9661, Cell Signaling), anti-Vimentin (chicken polyclonal, AB5733, Millipore), anti-Collagen I (rabbit polyclonal, ab34710, Abcam), anti-LTβR (rabbit polyclonal, ab70063, Abcam), anti-CD4 (rat monoclonal, GK1.5, BD), anti-CD11b (rat monoclonal, M1/70, BD), anti-CD11c (hamster monoclonal, N418, Bio-Rad), anti-Perilipin (guinea pig polyclonal, 20R-PP004, Fitzgerald), anti-PNAd (rat monoclonal, MECA-79, BD), anti-ICAM1 (rat monoclonal, YN1/1.7.4, Abcam), anti-Prox1 (rabbit polyclonal, 102-PA32, ReliaTech) and FITC- or Cy3-conjugated anti-αSMA (mouse monoclonal, 1A4, Sigma-Aldrich). FITC-, Cy3-, or Cy5-conjugated secondary antibodies were purchased from Jackson ImmunoResearch. Lipids were stained with BODIPY (Invitrogen) and nuclei were stained with DAPI (Invitrogen).

	The following antibodies were used for immunoblotting : anti-YAP (rabbit monoclonal, D8H1X, Cell Signaling), anti-phospho-YAP (rabbit polyclonal, 4911, Cell Signaling), anti-YAP/TAZ (rabbit monoclonal, D24E4, Cell Signaling), anti-TAZ (rabbit monoclonal, V386, Cell Signaling), anti-LATS1 (rabbit monoclonal, C66B5, Cell Signaling), anti-phospho-LATS (rabbit monoclonal, D57D3, Cell Signaling), anti-CTGF (rabbit polyclonal, ab6992, Abcam), anti-RelB (rabbit monoclonal, D7D7W, Cell Signaling), anti-CTGF (rabbit polyclonal, ab6992, Abcam), anti-p100/p52 (rabbit polyclonal, 4882, Cell Signaling), anti-p100/p52 (rabbit monoclonal, sc-7386, SantaCruz), anti-NIK (rabbit polyclonal, 4994, Cell Signaling), anti-LaminB (rabbit monoclonal, D4Q4Z, Cell Signaling), anti-GAPDH (rabbit monoclonal, D16H11, Cell Signaling), and anti-β actin (rabbit monoclonal, AC-74, Sigma-Aldrich).
Validation	All the antibodies used in this study were validated for the species and applications by the indicated manufacturers and our lab.

Eukaryotic cell lines

Policy information about cell lines

Cell line source(s)	Human FRC (primary culture), murine FRC (primary culture), MEF (primary culture), HEK-293T (ATCC), C3H10T1/2 (ATCC)
Authentication	Cells were authentificated based on their morphology, growth condition and specific gene expression.
Mycoplasma contamination	Cell lines were tested negative for mycoplasma contamination.
Commonly misidentified lines (See ICLAC register)	N/A

Animals and other organisms

Policy information about studies involving animals; ARRIVE guidelines recommended for reporting animal research

Laboratory animals	Specific pathogen-free (SPF) C57BL/6J mice, Rosa26-tdTomato mice, Rosa26-eYFP, Actb-DsRed, Actb-GFP, and OT-II mice were purchased from the Jackson Laboratory. Lats1flox/flox, Lats2flox/flox, Yapflox/flox , Tazflox/flox , Ltbrflox/flox9, Ccl19-Cre and Pdgfrb-Cre-ERT2 mice were transferred, established, and bred in SPF animal facilities at KAIST. All mice were maintained in the C57BL/6 background and fed with free access to a standard diet (PMI LabDiet) and water.
Wild animals	The study did not involve wild animals.
Field-collected samples	The study did not involve samples collected from the field.
Ethics oversight	Animal care and experimental procedures were performed under the approval from the Institutional Animal Care and Use Committee (No. KA2016-12) of KAIST.

Note that full information on the approval of the study protocol must also be provided in the manuscript.

Human research participants

Policy information about studies involving human research participants

Population characteristics	The bio-specimens and data used for this study were provided by the Biobank of CNUH, affiliated to the Korea Biobank Network.
Recruitment	To examine YAP and TAZ distribution in human LNs, several cervical LNs around thyroid papillary carcinoma were collected from the patients undergoing thyroidectomy with written informed consent according to the protocol approved by the institutional review board of Pusan National University (H-1610-002-003) and Samsung Medical Center (2018-06-061).
Ethics oversight	Pusan National University (H-1610-002-003), Samsung Medical Center (2018-06-061), and Chungnam National University Hospital (CNUH, 2016-10-037)

Note that full information on the approval of the study protocol must also be provided in the manuscript.

Flow Cytometry

Plots

Confirm that:

- [x] The axis labels state the marker and fluorochrome used (e.g. CD4-FITC).
- [x] The axis scales are clearly visible. Include numbers along axes only for bottom left plot of group (a 'group' is an analysis of identical markers).
- [x] All plots are contour plots with outliers or pseudocolor plots.
- [x] A numerical value for number of cells or percentage (with statistics) is provided.

Methodology

Sample preparation	Skin-draining LNs (axillary, brachial, cervical, and inguinal LNs) were harvested and cut into small pieces and digested in 2 ml of enzyme buffer containing 2 mg/ml collagenase type II (Worthington Biochem), 0.1 mg/ml DNase (Roche), and 1mg/ml dispase (Gibco) at 37°C for 30 m. Tissues were gently agitated and pipetted 1-2 times during digestion to disrupt any cell clumps. When LNs were completely digested, cell suspension was filtered through 40 µm nylon cell strainer and washed. Cells were incubated for 20 m with anti-CD45 Microbeads (Miltenyi). To enrich the stromal cell fraction, hematopoietic cells were depleted using AutoMACS (Miltenyi), according to the manufacturer's instructions.<br>For cell cycle analysis, mice were injected with 1mg of BrdU solution. Skin-draining LNs were harvested at 16 h after the injection of BrdU and isolated cells were processed with the APC BrdU flow kit (BD Biosciences) according to the manufacturer's protocols.
Instrument	Cell sorting was performed with FACSAriaII (BD Biosciences). The cell cycle profiles were analyzed with FACS Canto II (BD Biosciences).
Software	FACS DIVA and FlowJo software were used for data analyses.
Cell population abundance	Dead cells were excluded using DAPI staining (Sigma Aldrich). Sufficient cells were sorted and pooled for RNA-sequencing and analysis.
Gating strategy	Cell sorting/analysis:<br>1. CD45+ cells were pre-excluded by MACS or FACS.<br>2. FCS/SSC gating to discard debris, then DAPI/SSC to select alive cells.<br>3. CD31- PDPN+ FRCs were analyzed.<br>Cell cycle analysis:<br>1. CD45+ cells were pre-excluded by MACS .<br>2. FCS/SSC gating to discard debris, then DAPI/SSC to select alive cells.<br>3. Tomato+ FRCs or CD3ε+ CD19- T cells or CD3ε- CD19+ B cells were analyzed.<br>Following antibodies were used for flow cytomery:<br>Anti-CD45 (rat monoclonal, 30-F11, eBioscience), anti-TER-119 (rat monoclonal, TER-119, eBioscience), anti-PDPN (syrian hamster monoclonal, 8.1.1, Biolegend), anti-CD31 (rat monoclonal, MEC 13.3, BD), anti-CD3ε (hamster monoclonal, 145-2C11, BD), anti-CD4 (rat monoclonal, GK1.5, BD), anti-CD19 (rat monoclonal, 6D5, Biolegend), anti-CD8 antibody (rat monoclonal, 53-6.7, BD) and anti-IFN-g antibody (rat monoclonal, XMG1.2, BD).

☒ Tick this box to confirm that a figure exemplifying the gating strategy is provided in the Supplementary Information.
